# A Holistic Framework to Improve the Uptake and Impact of eHealth Technologies

**DOI:** 10.2196/jmir.1672

**Published:** 2011-12-13

**Authors:** Julia EWC van Gemert-Pijnen, Nicol Nijland, Maarten van Limburg, Hans C Ossebaard, Saskia M Kelders, Gunther Eysenbach, Erwin R Seydel

**Affiliations:** ^1^Department of Psychology, Health and Technology/Center for eHealth Research and Disease ManagementFaculty of Behavioural SciencesUniversity of TwenteEnschedeNetherlands; ^2^National Institute for Public Health and the Environment (RIVM)BilthovenNetherlands; ^3^Centre for Global eHealth InnovationDepartment of Health Policy, Management and EvaluationUniversity of TorontoToronto, ONCanada

**Keywords:** eHealth, design, participation, implementation, evaluation, multidisciplinary approach, Health 2.0, Wiki, e-collaboration

## Abstract

**Background:**

Many eHealth technologies are not successful in realizing sustainable innovations in health care practices. One of the reasons for this is that the current development of eHealth technology often disregards the interdependencies between technology, human characteristics, and the socioeconomic environment, resulting in technology that has a low impact in health care practices. To overcome the hurdles with eHealth design and implementation, a new, holistic approach to the development of eHealth technologies is needed, one that takes into account the complexity of health care and the rituals and habits of patients and other stakeholders.

**Objective:**

The aim of this viewpoint paper is to improve the uptake and impact of eHealth technologies by advocating a holistic approach toward their development and eventual integration in the health sector.

**Methods:**

To identify the potential and limitations of current eHealth frameworks (1999–2009), we carried out a literature search in the following electronic databases: PubMed, ScienceDirect, Web of Knowledge, PiCarta, and Google Scholar. Of the 60 papers that were identified, 44 were selected for full review. We excluded those papers that did not describe hands-on guidelines or quality criteria for the design, implementation, and evaluation of eHealth technologies (28 papers). From the results retrieved, we identified 16 eHealth frameworks that matched the inclusion criteria. The outcomes were used to posit strategies and principles for a holistic approach toward the development of eHealth technologies; these principles underpin our holistic eHealth framework.

**Results:**

A total of 16 frameworks qualified for a final analysis, based on their theoretical backgrounds and visions on eHealth, and the strategies and conditions for the research and development of eHealth technologies. Despite their potential, the relationship between the visions on eHealth, proposed strategies, and research methods is obscure, perhaps due to a rather conceptual approach that focuses on the rationale behind the frameworks rather than on practical guidelines. In addition, the Web 2.0 technologies that call for a more stakeholder-driven approach are beyond the scope of current frameworks. To overcome these limitations, we composed a holistic framework based on a participatory development approach, persuasive design techniques, and business modeling.

**Conclusions:**

To demonstrate the impact of eHealth technologies more effectively, a fresh way of thinking is required about how technology can be used to innovate health care. It also requires new concepts and instruments to develop and implement technologies in practice. The proposed framework serves as an evidence-based roadmap.

## Introduction

The impact of eHealth technologies is sometimes questioned because of a mismatch between the postulated benefits and actual outcomes. A lack of evidence about the distinct effects of eHealth technologies on health and health care is apparent [1-4]. Health care professionals are often skeptical and show little support for eHealth because technology does not seem to work for them or the benefit of their patients [5]. As a result, eHealth technologies often face adoption problems.

What could explain this mismatch? We know from research and the literature [1,2,4] that inadequate reimbursement and legislation can slow down the pace of innovation. Investors need to have trust before they can finance eHealth projects [2]. Apart from economic trust, a complex innovation needs coordination and communication [6], especially in the case of chronic disease management, where a variety of stakeholders are involved. Introducing eHealth technologies into the health care system requires careful coordination and communication among health care professionals, patients, informal caregivers, end users, and others. This is exactly what seems so hard to realize in practice. The same goes for project management; the precise definition of scope and objectives of the eHealth technology, the casting of participants, and the timely allocation of well-defined powers (eg, recourses and opinion leaders) and responsibilities are often not well defined beforehand. In day-to-day health care practice, these components are often present only on a superficial level, or not at all. In this situation, a lack of coordination and management deeply affects the outcomes from eHealth technologies research. Conversely, post hoc analysis does not, or cannot, account for the clouding of possible effects due to these important factors.

Another cause for the supposed low impact of eHealth technologies is the peripheral position of the users. eHealth technologies are often developed with only a marginal level of engagement from the (end) user. This lack of human centeredness explains the incidence of usability problems [7-9], or high attrition rates [10-18]. People simply stop using technologies that do not correspond in any way with their daily lives, habits, or rituals. In the end, the use of new technologies appears to be time consuming and frustrating for all those involved. In this way, technology-driven approaches result in “high tech-with-a-low impact” eHealth technologies [19-22].

All these confounding factors are not inextricably tied up with technology. Rather, avoiding them would reveal the real impact of eHealth technologies. The way in which technology is being designed to improve health care needs rethinking. The approaches that are being used to develop eHealth technologies are not productive enough to create technologies that are meaningful, manageable, and sustainable.

The development of eHealth technologies involves more than simply designing a product or service, and includes more than merely procuring stand-alone medical devices. We recognize the social dynamic and significance of eHealth technologies and their potential for improving health care. Creating a new technology often forces us to clarify how the process of health care delivery actually runs—for example, who the key stakeholders are and how payment is organized. It also illustrates the interdependencies between technology, people, their sociocultural environment, and the infrastructural organization of health care. Ideally, all stakeholders should be aware of these complex relationships [23].

In the wake of health 2.0 and medicine 2.0 initiatives [24, 25], a growing number of studies have emphasized the importance of a participatory development process involving (end) users—and other stakeholders such as payers, decision makers, insurers, and government officials—to increase the uptake of eHealth technologies [24]. Yet, in current perspectives on and definitions of health 2.0 [25], the role of stakeholders is not often addressed, nor is the potential of eHealth technologies to create infrastructures for better, cheaper, and easier-to-get health care services.

As long as the need to create a better fit between technological, human, and contextual factors continues to go unaddressed, the uptake and impact of eHealth technologies will remain at the very least poor, and at best undecided [4,26-28]. Therefore, we believe that a *holistic* approach is needed. Holistic means that we emphasize the importance of the whole and the interdependence of its parts, and avoid separate analysis of its parts. Such an approach would account for the issues of finance, management and technology when designing, implementing, and evaluating eHealth technologies. It constructs a productive fit through the integration of persuasive and human-centered design principles and business modeling. The urgent need for a holistic perspective to overcome the obstacles that stand in the way of the uptake of eHealth technologies has already been recognized [6,28].

The aim of this viewpoint paper is to boost the uptake and impact of eHealth technologies by advocating a holistic development approach. To this end, we undertook a critical appraisal of existing eHealth frameworks. First, we tried to identify the constituent elements of the framework: the target groups, the goals related to the development, implementation, and evaluation of eHealth technologies, the theoretical backgrounds, the visions on eHealth, and strategies or principles to increase the uptake and impact of eHealth technologies. In particular, we evaluated the extent to which the frameworks aim to realize a fit between human, organizational, and technological factors. Second, based on the outcomes of the review and supported by current knowledge on eHealth technologies development, we present the working principles for the holistic development process of eHealth technologies. And third, we build these principles into a holistic framework for developers, researchers, and decision makers. This holistic framework intends to guide the development of eHealth technologies. It already does so in three of our case studies in infection management, dermatology, and diabetes. The roadmap represents our current view on the development of eHealth technologies. It is a dynamic framework and we also publish it as a wiki for collaborative use (http://ehealthwiki.org).

## Review of Existing eHealth Frameworks

### Selection Procedure

We searched the literature through the electronic databases PubMed, ScienceDirect, Web of Knowledge, PiCarta, and Google Scholar. Journal indexes were searched. Examples of journals searched are the *Journal of Medical Internet Research*, *International Journal of Medical Informatics*, *Telemedicine and e-Health*, *Journal of Telemedicine and Telecare*, and *Journal of the American Medical Informatics Association*. Using a snowball and cross-referencing methodology, we included relevant cited and related articles.

We included eHealth frameworks based on the following inclusion criteria:

1. The paper must be published in a peer-reviewed journal.
2. The paper must either describe an eHealth theory, perspective, framework, or model, or contain a literature review. We particularly sought frameworks that provide a set of guiding principles for improving the development, uptake, and impact of eHealth technologies. A framework is considered as a set of (1) principles: assumptions, constructs, quality criteria, and ideas that guide research and development, and/or (2) strategies: hands-on guidelines, design heuristics, and methods to assist the development process, and/or constructs or criteria that have to be met to increase the quality of eHealth technologies (definition based on Kaufman et al [29]).
3. The proposed framework must propose quality criteria for the design, implementation, and evaluation of eHealth technologies and must account for multilevel factors of a human, technical, environmental, or organizational nature.
4. The title of the journal paper must include at least one of the following search terms: *eHealth* or similar terms, such as telemedicine, telecare, telehealth, health information systems/technology, or interactive health communication applications; AND *development* AND/OR *design*, AND/OR *implementation*, AND/OR *evaluation*, AND *framework*, AND/OR *quality*, AND/OR *success* (in terms of improved or innovated health care referring to cost benefits, health outcomes, behavioral outcomes, or care organization).

We identified 60 journal papers (see Multimedia Appendix 1) based on the search criteria used. Journal papers with a general focus that described merely the potential of eHealth (16 papers, general) were excluded from the analysis. We selected 44 for a full review. From these, we excluded those papers that did not describe a framework providing hands-on guidelines, or quality criteria for the design, implementation, and evaluation of eHealth technologies (28 papers, nonframeworks). We did not make any restrictions regarding the kinds of technologies used. From the results retrieved, 16 eHealth frameworks (see Table 1, Multimedia Appendix 1, Multimedia Appendix 2, and Multimedia Appendix 3) were identified that matched the inclusion criteria. Multimedia Appendix 1 shows the included and excluded eHealth journal papers.

**Table 1:** eHealth frameworks that matched the inclusion criteria.

Framework	Corresponding author
fr.1	Esser & Goossens [104]
fr.2	Catwell & Sheikh [23]
fr.3	Yusof et al. [28]
fr.4	Hamid & Sarmad [50]
fr.5	Pagliari [48]
fr.6	Kaufman et al. [29]
fr.7	Dansky et al. [6]
fr.8	Van der Meijden et al. [30]
fr.9	Shaw [27]
fr.10	Kazanjian & Green [49]
fr.11	Kushniruk [60]
fr.12	Hebert [33]
fr.13	Eysenbach [117]
fr.14	Eng et al. [51]
fr.15	Jai Ganesh [52]
fr.16	Kukafka et al. [26]

## Results

The first objective of the present review was to identify the strategies that are proposed for addressing the uptake and impact of eHealth technologies. Second, we wished to know how far these strategies draw on a holistic approach that strives to accomplish a fit between the human, organizational, and technology aspects. Since 1999, several eHealth frameworks have been published that describe a vision on how to increase the impact of eHealth technologies. Multimedia Appendix 2 presents the target groups and goals of the frameworks, the theoretical foundation, and definitions of eHealth technology that underpin the frameworks. Multimedia Appendix 3 presents the strategies and principles that are considered essential for the development of eHealth technologies, as well as the proposed evaluation methods.

### Target Group

To whom are the frameworks meant to be applied and who is involved in carrying out the tasks (development, research, and employment) that have to be accomplished? From Multimedia Appendix 2 (target groups), it is clear that the frameworks are aimed at different target groups. These target groups vary from *single* groups—designers (framework fr. 1, 2), decision makers (fr. 10), and health planners (fr. 16)—to *multiple* groups—researchers and others (fr. 7), researchers and practitioners (fr. 3), researchers and developers (fr. 5, 6), developers, health care providers, purchasers, consumers, and policy makers (fr. 14).

The frameworks are usually expert driven; that is, they are meant for experts such as designers or researchers. However, these target groups are rarely specified, so the type of design professional or researcher that belongs to them remains unclear. An exception to this rule are frameworks 5 and 10, where health service researchers (fr. 5) are targeted, as well as policy makers and administrative developers of information systems (fr. 10). Quite a few authors do not specify the target group that their frameworks (fr. 4, 8 ,9, 11–15) are supposed to serve.

Six of the frameworks (fr. 3, 5 , 6, 7, 10, 14) address multiple target groups, although it is not clear what kind of tasks the different groups have to carry out in the subsequent development process. These frameworks provide, for instance, guidelines for evaluation as part of the development process. But it is not clear who is responsible for what kind of task. No specification has been provided for those (by discipline) who are involved in *producing* the eHealth technology and those who are involved in the *deployment* of the eHealth technology.

### Goals

What do the authors want to achieve with the proposed frameworks? As shown in Multimedia Appendix 2, all of the frameworks aim to improve either the *uptake* (eg, implementation and adoption of eHealth technologies) or the *impact* (eg, effectiveness of eHealth technologies), or both. The frameworks are supposed to assist the target groups in the development of eHealth technologies via checklists, guidelines, and criteria. Frameworks that aim to enhance the uptake of eHealth technologies (fr. 1, 3–5, 7, 8, 10, 13–16) can be used for *formative (process-driven) evaluation purposes* to assess user acceptance and satisfaction, widespread adoption, or implementation (eg, infrastructure and resources) of eHealth technologies. The frameworks that aim to enhance impact (fr. 1–3, 5–8, 12, 14) can be used for *summative (performance-driven) evaluation purposes* to assess the potential of eHealth technologies to enhance the quality of health care, benefits, performance, and effectiveness (eg, health outcomes and cost reductions). Two frameworks (fr. 8, 12) aim to enhance the *success* of eHealth technologies. The term success is used by the authors in different ways. Van der Meijden et al [30] (fr. 8) refer to the six dimensions of success defined by DeLone and McLean [31,32]: system, service and information *quality*, to user *acceptance*, and individual and organizational *impact.* Hebert [33] (fr. 12) refers to Donabedian’s [34] quality-of-care measures: structure, process, and outcome. The frameworks that aim to increase both the uptake and impact (fr. 1, 3, 5, 7, 8, 14) have more potential to create a perfect fit between human, organizational, and technology factors. Remarkably, only two frameworks guide the decision-making process via scientific evidence (fr. 2, 10), while one channels the investment of eHealth technologies (fr. 12). Some frameworks (fr. 9, 11) do not explicitly state any goals.

### Foundation

What theories or models underpin the frameworks? What empirical evidence is this grounded in? The frameworks are based on a combination of models, theories, and literature review studies (fr. 1–5, 8, 10–13, 15, 16), and some are validated by experts (fr. 1) or tested via empirical research (fr. 3, 9, 12). The authors of the frameworks (fr. 4, 16) argue that the development of eHealth technologies should be grounded in multidisciplinary theories such as behavioral and sociocognitive theories and those linked to innovation and diffusion. Two frameworks are based on regulations (the Health Insurance Portability and Accountability Act [6], fr. 7) and institutional regulations that make health communication programs work (National Cancer Institute [35], fr. 14).

The theoretical foundations of current frameworks stem from *human–technology interaction* models based on software engineering principles and behavioral theories; *health service* models for quality of health care; and *innovation diffusion* models (Multimedia Appendix 2: foundation). Given the complexity of health care, some authors argue that more contingency-driven models are needed to address the organizational and environmental aspects that influence the uptake of eHealth technologies: sociotechnical and contextual aspects (fr. 2), the IT-Organization Fit model (fr. 3), diffusion theories (fr. 4), health services evaluation methods (fr. 5, 10, 12), and cross-theoretical integration of behavior models (fr. 16).

The *human–technology interaction* models (fr. 1–6, 8, 11, 16) are aimed at achieving user centeredness for eHealth technologies, which is considered to be a prerequisite for the acceptance of eHealth technologies in practice. Examples are software design models [36-38], information system success models [31], and technology acceptance models [39-41]. These models focus on the factors that influence usability, acceptance, or adoption of eHealth technologies. Framework 1 is a *human–media interaction* framework based on the media richness theory to support the patient–caregiver interaction (based on Miller [42]).

Frameworks 12 and 13 are based on *service-quality* models such as Donabedian’s [34] quality-of-care measures. Health technology assessment (fr. 12) was used as an approach to assess the value of eHealth technologies in practice, and in the World Health Organization strategies for (re)designing health care systems (fr. 14).

The frameworks that highlight the contextual aspects that are important for the integration and operation of eHealth technologies in the health care context are founded in *innovation diffusion* models such as the Precede-Proceed Model (fr. 16), social-cognitive theory, diffusion of innovation theory (fr. 15, 16), IT-Organization Fit Model (fr. 3), and acts/legislation such as the Health Insurance Portability and Accountability Act (fr. 7, 10).

Through literature reviews we identified aspects that influence the success of eHealth technologies (fr. 8, 13) and aspects that are critical for evaluation during the development of eHealth technologies (fr. 2–6, 10, 12, 16). The results were used to ground the development approach (stages from ideation to rollout) or to formulate criteria for the evaluation of research activities related to the development of eHealth technologies. Empirical evidence for the frameworks stem from expert validation (fr. 1) to assess the relevance of the frameworks or from pilot testing in practice (fr. 3, 9, 12) to assess the utility of the framework.

### eHealth Definition and Technology Focus

What definitions of eHealth were used as a basis for the frameworks? What kinds of technologies did the frameworks focus on? Some authors use their own definitions of eHealth (fr. 1, 5, 8, 15; see Multimedia Appendix 2). Framework 1 uses a definition for telemedicine and refers to the use of information and communication technologies for the exchange of medical information in a clinical setting, aimed at a specific technology, teleconsultation. Framework 5 refers to medical informatics and health services research as a synonym for eHealth, related to health technology assessment and health systems research. The definition of framework 8 refers to health information systems: general patient care information systems in hospital settings or specific care information settings (intensive care unit). Framework 15 defines eHealth as the use of electronic information and communication technology to promote health or improve health care. The authors (fr. 15) explicitly mention that the infrastructure of an eHealth program consists of three components: human, technical, and medical.

The other definitions come from researchers in the field of eHealth. These definitions are in a certain way related to the technologies the frameworks focus on. For example, the definition from the Institute of Medicine is used (fr. 10) to classify health information systems and the definitions of telehealth by Field [43] and Reid [44] are used to describe the use of technologies in rural areas or in cases where social or cultural barriers separate the participants (fr. 12). Four frameworks (fr. 2, 4, 7, 14) have a wider focus referring to multiple technologies and modalities for the organization and delivery of health care services and information. Framework 2 [23] used the definition of Eysenbach [45] (see Multimedia Appendix 2). The authors of framework 2 argue that “...the definition of eHealth should encompass the full spectrum of ICTs [information and communication technologies], whilst appreciating the context of use and value they can bring to society” [28] is aimed at better service utilization via eHealth technologies in general. It uses a description of eHealth from Canada’s Health Informatics Association (defined in Oh et al [46]) to connect providers, patients, and governments; to educate and inform health care professionals, managers, and consumers; to stimulate innovation in care delivery and health system management; and to improve the health care system.

The authors of framework 7 expand on the evolution of eHealth, changing from a one-way system to wireless technologies and online communities using Web 2.0 technologies. The authors state that eHealth is revolutionizing health care, resulting in new models for eHealth development:

eHealth has moved from an acute-care orientation to prevention and disease management, from an individual focus to a population focus, and from an institutional setting to communities and cyberspace. Concomitantly, models of healthcare delivery have evolved from being physician and clinician driven, to patient-centered care models that are based on participative decision-making [6].

Framework 14 uses a definition from Robinson et al [47] for interactive health communications, which is the focus of their framework (see Multimedia Appendix 2). They posit that they do not focus on eHealth technologies that focus exclusively on logistics or clinical data. In some cases, no definitions on eHealth were reported (fr. 3, 6, 9, 11, 13, 16).

### Strategies and Principles for eHealth Research & Development

The frameworks propose different strategies and principles for the development of eHealth technologies (presented in Multimedia Appendix 3). Almost all of the frameworks plead for a *multidisciplinary development approach*, *continuous and systematic evaluation* during development, and *robust methods* for formative and summative evaluations to realize technologies that are aligned with the needs of their users and environmental aspects.

#### A Multidisciplinary Development Approach

Several frameworks (fr. 2–5, 7, 9, 10, 14–16) posit that a *multidisciplinary development approach* (see Multimedia Appendix 3) is important when developing eHealth technologies. A multidisciplinary approach is considered as the involvement of different disciplines in the development of eHealth technologies referring to a team of various experts that guide the development, or the involvement of various stakeholders that can be affected by the use of the eHealth technologies.

Some frameworks advocate a user-centered design approach that takes into account the needs of the end users (patients and/or health care providers) during the development process (fr. 1, 4, 6, 8, 11–13). Others propose a comprehensive overall approach that addresses the importance of involving different stakeholders (patients, clinicians, managers, information technology providers, the health care organization, etc) in the development process (fr. 2, 3, 7, 10, 14–16) in order to document the complex relationships between political, social, organizational, and technical worlds (fr. 2); ensure that different contexts and visions are taken into account (fr. 3, 7, 9, 10); identify the values and concerns different stakeholder have (fr. 16); or develop sustainable eHealth technology programs (fr. 15).

Pagliari [48] (fr. 5) and Kazanjian and Green [49] (fr. 10) argue that a multidisciplinary development *team of experts* is needed to maximize the potential of eHealth. Such a team should consist of a “wider constituency of disciplinary experts including social, management, and legal scientists, all of whom have a stake in the field” (fr. 5) and “a number of disciplinary perspectives, incorporating theories of epidemiology, sociology, economics, and systems science; and applies critical theory to health care evaluation” (fr. 10). Dansky et al [6] (fr. 7) state that a multidisciplinary team is needed to organize the development process to identify the staff and skills needed to implement eHealth technologies, and that roles and responsibilities should be identified in order to organize the research (data collection) and communications (involving stakeholders).

The participation of *stakeholder*s is viewed as essential to reflect on the values, drivers, and goals of the eHealth technologies to be developed. For example, Catwell and Sheikh [23] (fr. 2) argue that design teams need to have a thorough understanding of the stakeholders’ needs, concerns, values, and beliefs, and define what the eventual system will be expected to provide:

The rich picture of the real world needs to be developed into a conceptual model so that stakeholders can reflect critically on the drivers, vision, and goals of the project and agree whether such a program of change is appropriate and feasible...It is important that the initial elicitation stage goes beyond functional and technical requirements and considers for example, accessibility, acceptability, and affordability issues [23].

Hamid and Sarmad [50] (fr. 4) state that multiple stakeholders should be involved in the evaluation process. They state that one stakeholder, or a group with a common perspective, is the most important to be addressed: the user. This is a different view from the other frameworks in so far as other frameworks argue that a multiperspective view is needed to ensure that eHealth technologies fit with their users and other contingent variables (culture, organizational needs, etc). Eng et al [51] (fr. 14) refer to the statement of the Science Panel on Interactive Communication and Health, namely that four stakeholder groups should participate in order to improve the quality of eHealth technologies: consumers (patients, families, and caregivers), health care professionals and purchasers, developers, and policy makers. They argue that these stakeholders should participate at an early stage of developing the applications “to increase the probability of a favorable impact on health and quality outcomes.” Jai Ganesh [52] (fr. 15) posits that a multidisciplinary collaboration is needed to increase acceptance (by consumers/patients and health care professionals) and “to establish joint ventures in the field of eHealth by inviting local or foreign partners to participate and to take equity stakes in the delivery of eHealth services.” In this approach, it is important “to identify appropriate partners to specify appropriate technology and to find financing.” The key players that should collaborate are the patients, practitioners (health care professionals), and health care service providers (see Multimedia Appendix 3). The aim of the collaboration is to bring together information technology experts, health professionals, lawyers, industry representatives, and others to ensure sustainable eHealth technologies.

Kukafka et al [26] (fr. 16) promote active participation via a participatory design approach to ensure that planners have a “structure in place to engage system end-users effectively from the start.” They state that end users, management staff, and administrators should all be engaged in diagnosing the problems. “This process enables planners to expand their knowledge of the organization by identifying the values and subjective concerns key stakeholders have with existing systems and procedures.” The authors do not specify what kind of stakeholders should be involved to facilitate multidisciplinary collaboration.

The authors of the frameworks that argue for a multidisciplinary development approach have different views on who should participate in the development process and what is actually meant by a multidisciplinary approach. The frameworks describe the actors that should participate in the development process either in terms of *disciplines*—clinical, human, social, organizational (environment), administrative (logistics), technical (industry), and political—or in terms of *stakeholder groups*—technology developers and health service researchers, clinicians/health care providers, payers, purchasers, policy makers, lawyers, and consumers/end users (patients, families, and caregivers).

#### Continuous and Systematic Evaluation

Multimedia Appendix 3 shows that some frameworks (fr. 2, 5, 6, 8, 11, 14) explicitly promote a continuous and systematic evaluation throughout the development of eHealth technologies to ensure that eHealth technologies are truly user informed, fit for context, of high quality, and of demonstrated value (fr. 5). These *process-driven* frameworks describe a cyclic, iterative research evaluation and development approach. The process starts with identifying the needs and goals of the intended users or stakeholders, compiling a requirements analysis of the design, prototyping, and implementation. Each stage is accompanied by its own set of evaluation research activities. For example, framework 5 provides different evaluation phases for research (formative and summative) from concepts to rollout. According to the author, there is a growing acceptance that evaluation should ideally be approached as a longitudinal process occurring through a series of overlapping and iterative stages relevant to the maturity of the technology in its life cycle, from initial conception through rollout. Framework 11 presents formative and summative evaluation phases as well, from project planning to implementation. The authors of framework 14, Eng et al, state that evaluation should be woven throughout all stages of the development process: conceptualization (formative evaluation; needs driven), design (prototyping), implementation and dissemination of product development (process evaluation of operationalization), and outcome evaluation (refinement). Evaluation is seen as crucial for the development of eHealth technologies, and the research activities for formative evaluations are related to each stage of the development process. Formative evaluation is considered to be a central iterative research activity that should be initiated during the early stages of development in order to assess the problems and needs of the various stakeholders. Van der Meijden et al [30] (fr. 8) argue that evaluation is often aimed at measuring the effects (summative), while neglecting the value of formative or process evaluation to improve a technology during development and implementation. Evaluation, in their view, should start *before* the technical development and has no fixed end because the technology fluctuates over time. The evaluation should include “multiple, selected periods of data collection and all stakeholders’ points of view.”

Frameworks 1, 3, 4, 7–10, and 12–16 can be considered as *quality-assessment frameworks*. These frameworks provide quality-assessment criteria, or evaluation dimensions and factors for the design, implementation, or success (ie, impact) of eHealth technologies. The authors of frameworks 3, 7, 9, and 16 state that it is important to address all aspects of health care that can be influenced by the use of eHealth technologies to ensure the fit between the technology, its users, and all of the organizational aspects. The evaluation dimensions influence each other in a temporal and causal way; this means that *fit* can be viewed in terms of strategic planning and strategic alignment (managing technology with organizational needs), as well as in terms of a fit between human and organizational needs and alignment between human needs and technology.

In fact, all of the quality-assessment frameworks address in a certain way contextual or environmental factors. Framework 1 presents criteria for teleconsultation specified to its users, the patient–provider communication, and the compatibility with the organizational context. Framework 4 is aimed at user centeredness, providing criteria for the fit between the system and its users, and the system’s fit with the health care system. Framework 16 proposes critical assessment phases to determine organizational needs and goals amenable to technology (information technology solutions) and factors that influence human behavior. According to Yusof et al [28] (fr. 3), an evaluation of human, organizational, and technology aspects is required throughout the whole development cycle of planning, analysis, design, implementation, and operation and maintenance.

#### Robust Methods for Evaluation

The authors of several frameworks (fr. 2–6, 8–11, 14) argue that more rigorous evaluation approaches or methods are needed to assess the added value of eHealth technologies (see Multimedia Appendix 3). A mixed-methods approach is often proposed, combining qualitative methods (such as observations and interviews) with quantitative methods (workflow sampling or questionnaires) (fr. 2, 3, 5, 6, 8, 9, 11, 14). Van der Meijden et al [30] (fr. 8) argue that the integration of qualitative and quantitative data collection methods provides an opportunity to improve the quality of the results through triangulation, as the data from different sources complement each other to provide a more complete picture. “The integration of qualitative (observations, interviews) and quantitative (questionnaires, work sampling) data collection methods provides an opportunity to improve the quality of the results through triangulation” [30].

Formative process evaluation measures (fr. 2, 6) and longitudinal process studies (fr. 5) are recommended to demonstrate the acceptability and utility of new eHealth technologies, as well as the conditions for implementation that may influence their adoption. Summative methods are mentioned to evaluate the validity and efficacy of a system, such as randomized controlled trials (RCTs) (fr. 5, 6), or the overall impact (fr. 9, 11). The authors of frameworks 5 and 9 state that methods should go beyond the use of RCTs to evaluate the impact of eHealth technologies, because RCTs are seen as less well suited to evaluate the impact of eHealth interventions in a complex environment or to study the effect they have on the care delivery process. “An eHealth technology is not a drug and should not be evaluated as such,” in Shaw’s view [27] (fr. 9).

Catwell and Sheikh [23] (fr. 2) are of the opinion that “formative iterative evaluations using simple prototypes of the eHealth technology may be useful during the early stage of the development process to assist with communicating ideas, building a common understanding, agreeing to objectives, and securing stakeholder buy-in.” Except for usability and prototyping instruments (fr. 2, 11), think-aloud methods for assessing usability (fr. 5), and the multiple methods presented by Yusof et al [28] (fr. 3) and Eng et al [51] (fr. 14), none of the frameworks present practical evaluation tools that are appropriate for the participation of various stakeholders in the development of eHealth technologies.

### Potentials and Limitations of the Reviewed Frameworks

#### Potentials

The reviewed frameworks provide useful insights for the development of eHealth technologies that are user centered and fit for context: they provide process guidelines and indicators for creating eHealth technologies that are acceptable, affordable, and manageable. The added value of the frameworks lies in the multifactor approach: combining factors that support the adoption and implementation that are often underestimated in research (eg, fr. 8, 9, 16).

The integration of different models or theories is another added value, to ensure that eHealth technologies are feasible and sustainable. Furthermore, the comprehensive and integrative approach of some of the frameworks (eg, fr. 3, 7, 9, 15) are of interest to widen the contribution of eHealth technologies to innovate health care and to reduce societal problems (eg, aging or costs).

Most frameworks advocate a multidisciplinary development approach, involving collaboration among different stakeholders (eg, payers, technology providers, patients, and health care professionals) and multiple methods (quantitative and qualitative) for assessing the process of technology development (formative evaluation) and the effects of eHealth technologies on health care (summative evaluations). One of the challenges expressed by the authors is the move from evaluations focusing exclusively on measuring outcome variables (via RCTs) to evaluations involving in-depth process data about the usage of the eHealth technologies in different care settings (hospitals or home-based care).

#### Limitations

##### Target Group

From the review we can conclude that the current frameworks have certain limitations. It is often not clear whether the frameworks are conceptual thoughts to provide insights and knowledge about the development of eHealth technologies or to use as a debating tool among researchers and others; or whether the frameworks should function as a practical guideline to assist developers. If target groups are reported, then it is not clear what kind of roles, tasks, or responsibilities these groups have during the development process. Another point is that the target groups are not specified. What kinds of disciplines or professions are representative of the researchers, designers, and other target groups? What level of cooperation is supposed between the different disciplines such as researchers, designers, and technical developers? And who is involved in the various stages from ideation to maintenance?

##### Goals

The frameworks aim to bring about the widespread diffusion and adoption of eHealth technologies, the implementation of eHealth technologies, or the improvement of the performance and effectiveness of eHealth technologies. It is often not clear what is meant by success, effectiveness, or performance. To realize the goals, different strategies and principles are presented. One of the concerns refers to the benefits or drawbacks of the frameworks, given the aims they strive for. It is unclear what the frameworks contribute toward increasing the uptake of eHealth technologies or innovating health care considering the wider social, political, or economic impacts of improvements in goal attainment.

##### Foundation

Evidence for the frameworks is based on scientific research or on literature reviews. A few empirical studies have been reported that help to ground the framework or demonstrate the benefits or drawbacks of the framework. It is often unclear how the theories or models that underpin the frameworks match the strategies reported. In general, the relationship between the visions on eHealth, proposed strategies, and research methods (formative and summative) are obscure, perhaps due to a rather conceptual approach that focuses on the rationale behind the frameworks rather than on practical guidelines.

##### eHealth Definition and Technology Focus

The frameworks address, in most cases, the evaluation of technologies that have already been defined (such as teleconsultation or health information systems), except for a few that do not focus on any specific form of technology in particular. The discussion about how to track down information about what kind of technologies (content or format) fit best with stakeholders’ needs and values and care settings is not yet underway. Another limitation is the bias toward information systems rather than social or safety technologies. The use of social media for cocreation is beyond the scope of the frameworks.

##### Strategies and Principles for eHealth Research & Development

Although the importance of a multidisciplinary approach during the development of eHealth technologies is acknowledged by most frameworks, only a few authors have actually worked on incorporating this into the strategies for research and development within their frameworks. The frameworks propose stakeholder involvement during the development process, as part of the evaluation cycles. Most of the frameworks do not provide any insights into the identity of the stakeholders, sometimes referring to the end users, sometimes to developers and others (eg, health care professionals, providers, and government) that have a stake in the development of eHealth technologies. Referring to Yusof [28], it is not clear who participates (which stakeholders’ perspective is going to be evaluated), when participation is needed, and by whom (at which phase in the system development life cycle), what the focus of the participation is (aspects or focus of the evaluation), and how participation can take place (methods of evaluation). In addition, the division of tasks between developers and stakeholders is not concise. In our view, the involvement of stakeholders is not restricted to the evaluation but extends to the full development process. Their involvement is important from ideation to the validation of business models.

Although the lack of contingency variables and the dominant focus on summative evaluations is reported as one of the main shortcomings of earlier models or frameworks, the current frameworks address the importance of contextual factors, but they do not incorporate these factors systematically into the frameworks’ strategies. In fact, implementation is not considered as being interwoven within the development of eHealth technologies. Notably, no models are mentioned for collaborative development (ie, participatory development, cocreation, or value creation), incremental development, and sustainable implementation (business modeling). The critical point is that no clear information is given about the periods or the roles and responsibilities of different stakeholders in several stages of the development process, or about the focus of evaluation regarding the involvement of various stakeholders, or the methods for participation.

The authors of the frameworks argue for more rigorous evaluation methods that go beyond the use of experimental designs (RCTs) to evaluate the impact of eHealth technologies, because RCTs are less well suited to evaluate the impact of eHealth interventions in a complex environment. Rigorous qualitative studies combined with quantitative and process evaluation measures are recommended; the instruments that accompany this, however, are rarely reported. The participation of users and stakeholders is considered important, but the methods and instruments needed to guide this participatory process are missing. The frameworks prescribe what should be done, but do not point to the instruments or tools to realize it. In fact, the greatest limitation of the frameworks is the lack of clear handles to support the development process; although the authors posit in their description of the frameworks the essential criteria for that, they did not translate this into their frameworks. For example, collaboration between the developers and the researchers is recommended, but no guidelines or prescribed activities are available for managing this type of development collaboration.

## A Holistic Framework for the Development of eHealth Technologies

We believe that a comprehensive view on supporting health care by technology is needed to ensure that eHealth technologies are used effectively and efficiently. That is to say, that they realize their objectives and do so with optimal use of resources (time, money, and staff). We propose a holistic approach for the development of eHealth technologies. It is based on the outcomes of the reviewed frameworks, on empirical research, and on progressive insights obtained from discussing the framework with researchers (eHealth conferences).

Holism maintains that properties of individual elements in a complex system are taken to be determined by the relations they bear to other elements [53,54]. When applied to social theory this means that “each term owns its meaning to its relations with the others, so that they are all more or less closely inter-defined, and a change in the meaning of one term will have repercussions for all the rest” [55]. A holistic perspective on eHealth technologies has been advocated elsewhere, for instance by Dansky et al [6], Yusof et al [28], or Kukafka et al [26].

Without addressing the full range of factors, strategies to change behavior run the risk of being ineffective because they fail to recognize interdependencies between individual and organizational factors [26]

For us it means that human characteristics, socioeconomic and cultural environments, and technology are considered to be closely connected to each other. eHealth technologies affect people in their daily lives. People always bring in their psychological makeup, their rituals and habits, and their social skills, which affect their personal and professional environment. This evidently affects their ability to interact with technology.

### Strategies and Principles for a Holistic Development Approach

**Figure 1:**
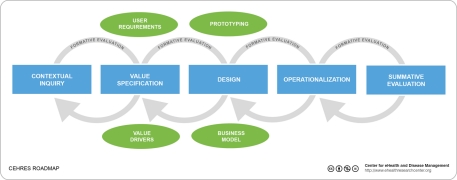
CeHRes Roadmap for the development of eHealth technologies.

We introduce six working principles derived from the review of current frameworks, as well as from empirical research [7-9,14,21,56]. These principles are the groundwork for a holistic framework for the development of eHealth technologies. The framework and its related concepts are presented in Figure 1.

#### 1. eHealth Technology Development is a Participatory Process

EHealth technology development is a matter of cocreation; stakeholder participation is essential [25]. Stakeholders’ involvement spans the full development process, starting from contextual inquiry and ending with summative evaluation (see Figure 1). Stakeholders can be considered as actors that have different roles in the development of eHealth technologies, from ideation to operationalization. Through their roles in identifying needs, or specifying critical issues for design and implementation, they help to create the technology [57,58]. Adequate project management needs to arrange for the participation of stakeholders and to identify their roles, tasks, and responsibilities.

#### 2. eHealth Technology Development Involves Continuous Evaluation Cycles

Development is an iterative, flexible, and dynamic process resulting in concepts of the technology (from ideation to prototypes). These concepts need to be evaluated continuously. Evaluation as such is a cyclic, longitudinal research activity interwoven with all stages in the development process and as such without a fixed end (formative and summative evaluation; see Figure 1). By its usage, technology is reshaped. In the case of interactive technology, users reflect on the content or system via feedback [59]. So, while technology evolves into action, evaluation follows on as a continuous, reflexive process that leads to matching human, organizational, and technology factors. This is noted in several of the reviewed frameworks [23,29,48,51,60]. Such evaluation takes place via formative cycles (see Figure 1) to reflect on the development process. Reflection is important to check for tacit understanding [61]. Stakeholders (including users) provide feedback and forward comments during the process [59]. They monitor their usage from their perspective.

#### 3. eHealth Technology Development is Intertwined With Implementation

Implementation is often seen as a postdesign activity. In our view, the conditions for implementation must be taken into account right from the start (contextual inquiry and value specification; see Figure 1) [62]. Potential implementation issues such as limited resources (eg, time, staff, and money) or personal drawbacks (eg, skills, motivation, and anxieties) should be identified [63-66]. These issues should also be accounted for in the subsequent stages (design and operationalization; see Figure 1). In this way, the well-known pitfalls of stakeholder disregard can be avoided.

#### 4. eHealth Technology Development Changes the Organization of Health Care

The development of eHealth technology in itself can be considered as the creation of new processes and infrastructures for health care delivery. It may reshape health care since it intervenes with traditional care characteristics such as the division of labor, or time- and place-dependant delivery [67]. This can be observed in today’s shift from hospital-based care to home-based care, which requires new reimbursement and planning systems. Though underestimated in current research, this catalyst effect is inherent to eHealth technology development.

#### 5. eHealth Technology Development Should Involve Persuasive Design Techniques

More and more patients wish to use technology for communicating and sharing personal information. They expect self-care technology to show understanding, to persuade them to do the right thing, or to provide rewards and appraisal for appropriate behavior [7,22]. However, the inherent capacities of technology as a persuasive medium for behavior change, information sharing, or self-management are often overlooked. Also, eHealth technologies often do not fit with the intended users’ needs [7-9,14,68-70]. Particularly in the context of long-term care, it is important to develop technologies that can create bonding relationships with the end users. Motivation and social support are functionalities of eHealth technologies that belong to the most important persuasive drivers [20]. Via persuasive techniques, eHealth technologies can be designed to match user profiles, and to motivate or inspire patients to engage in self-management [71-75] (design; Figure 1).

#### 6. eHealth Technology Development Needs Advanced Methods to Assess Impact

Several authors [1,2,4,6,62,76-87] note that a broader view is needed to assess the overall impact on health care. Both benefits and drawbacks have to be taken into account in terms of risks, ethics, performance, finance, or adherence. Impact is understood to evolve from an interaction between the technology, the person, and the context of usage. Interactions may be planned to be effective, to realize the outcome of the process. The process itself may be intended to be efficient, to use relatively few resources to achieve an objective. However, the constituents of this interaction are interdependent and mutually affect each other in a fuzzy manner: they may miss the target or take the hardest road. A holistic approach brings these elements together and targets the dynamics toward a desired, temporary situation that in the end is greater than its parts. Impact fluctuates over time and situations [30,88-90]; therefore, we need to have more advanced methods to assess the added value of eHealth technologies for health care and society. We need to understand *what* differences eHealth technologies can make in health care, *why* eHealth technologies make these differences, and *why* eHealth technologies may not have the expected impact [85,86,91]. Research on the impact of eHealth technologies is often done in clinical studies, mainly RCTs. This “gold standard” is often not suitable for identifying what works in practice. There are simply too many confounding factors that cannot be controlled or anticipated [83,92,93]. Moreover, impact in terms of organizational innovation cannot be measured in an RCT. For an RCT a certain degree of reduction is needed. This is exactly what influences the sustainability or effectiveness to be measured. Given these downsides, generalizing from their outcomes is at the least problematic. Some authors [30,48,60,80,84,85,94] have expressed the need for mixed methods using both quantitative and qualitative designs in order to better measure the uptake and impact of eHealth technologies.

The challenge lies in the integration of data collection from multiple sources, using a mixed-methods research design [95]. This implies a periodic evaluation during development rather than a before-and-after design, and advanced methods focusing on examining process variables (usage/dropouts of eHealth technologies) with methods measuring outcome variables (costs, health condition, or adherence to technology and interventions). The methods and instruments from the holistic framework will be described in a subsequent paper.

### Holistic Framework: CeHRes roadmap

#### Framework, Target Group, and Goal

To visualize and outline our holistic approach we have created a framework and have presented a CeHRes roadmap (Figure 1). This roadmap serves as a practical guideline to help plan, coordinate, and execute the participatory development process of eHealth technologies. The framework is meant for developers (eg, technicians, designers, and health care professionals), researchers, and policy makers and for educational purposes (eg, students and health care providers). It also serves as an analytical instrument for decision making about the use of eHealth technologies. 

#### eHealth Definition and Technology Focus

For the purpose of this paper, the term eHealth, or electronic health, refers to all kinds of information and communication technology used for supporting health care and promoting a sense of well-being. Within eHealth, a broad spectrum of technologies is used. These technologies include *Internet technologies*, such as informational websites, *interactive health communication applications* (ie, e-consultation, online communities, online health decision-support programs, and tailored online health education programs), *online health care portals*, and *electronic health records*. It also includes *mobile health communication programs*, and other advanced technologies such as *virtual reality programs* (ie serious gaming to stimulate exercise or 3-dimensional applications for the treatment of anxiety disorders), *home automation (domotics)*, sensor technology for independent living and remote monitoring, and *robotics*, the deployment of robots for assisting people with domestic tasks or to perform surgery.

The framework does not focus on the development or redesign of a specific technology; it should be used for all kinds of eHealth technologies with a scope broader than merely monitoring for medical purposes. In fact, our ultimate goal is to realize 5M-eHealth technologies that can support measurement (e-diagnose), monitoring (observation), mentoring (nudging), motivation (support), and management of data (automated integration of different data).

#### Foundation

The foundation of the framework is based on the aforementioned principles, reviews in the field of eHealth [2,3,22,96-99], progressive insights from eHealth research (see Multimedia Appendix 4), and multidisciplinary theories from psychology, communication, and human–computer interaction design.

In our view, integrating *persuasive technology design*, *human-centered design*, and *business modeling* provides the theoretical background for the development, evaluation, and implementation of eHealth technologies. As indicated by the authors of the frameworks that we reviewed, the development approach of eHealth technologies should be multidisciplinary in nature. Persuasive technology is the covering concept, referring to the use of technology to change people’s attitudes and behavior [71-75]. The conception that technologies, especially interactive technologies, can persuade people to do the right thing at the right moment is rather new in the health care domain. Technology, for example, can simplify or guide people through a process of self-care management or provide social support through tailored feedback. In the domain of eHealth, we think that research into persuasive design techniques is needed to understand how technology can motivate or inspire healthy behaviors, how the technology fits with the needs of users (human-centered design) [68,100-104], and how technology can create new structures for health care delivery.

In addition, the participation of stakeholders, such as caregivers, insurers, or decision makers, influences the development of eHealth technologies. Their needs, concerns, values, and beliefs determine what the eventual technology should provide in order to realize the goals. To understand and guide the value-creation process in order to develop eHealth technologies that are affordable and interoperable with the health care system, innovation models are needed. Jai Ganesh [52] states that eHealth programs should be based on sound economic frameworks to deliver value for the investment of eHealth technologies. eHealth technologies require substantial financial investment [105]. The business case for eHealth technologies depends on the expectation of a return on investment. Nonetheless, we should focus not only on value in terms of money, since eHealth technologies may have value for life. *Business modeling* originated in commercial strategic management [61,106-109], focusing on the collaborative efforts of value creation with stakeholders. Stakeholders, the ones who affect or are affected by eHealth technologies [57,58], reflect on each other’s values and weigh the importance of the values in terms of economic, behavioral, and psychological interests. This results in business models for the implementation of eHealth technologies. Concepts and techniques from business modeling help to identify critical factors for the implementation.

### Research and Development Activities

Figure 1 depicts the development process and accompanied research activities. The research and development activities will be explained below.

#### Multidisciplinary Project Management

Ideally, the development process of an eHealth technology should start with multidisciplinary project management. Multidisciplinary project management facilitates the cooperation between those who are responsible for *producing* the technology (eg, technicians, designers, and health care professionals) and those who *participate* to ensure that eHealth technologies fit in with the needs and values (eg, end users and health care insurers/payers) [110]. Project management is needed to avoid a design-build-run-and-see-what-happens approach. We see development as a cyclic process of ideation, designing, building, and evaluating a technology. Consequently a *multidisciplinary team of researchers and developers* (designers, technicians, health care professionals, and health care researchers) must guide the project management and conduct the *planning* in time and space [48]. Project management also requires logistical planning of how, when, and with what purpose *stakeholders* should participate in the research and development [57,58,110].

#### Contextual Inquiry

Contextual inquiry entails information gathering from the intended users and the environment in which the technology will be implemented. Field observations and interviews with the intended users are needed to obtain insights into the users’ day-to-day rituals and habits and how technology can be matched to that. Through workshops, stakeholders (including users) should be invited to discuss the problems and needs and the goals of the eHealth project via personas and scenarios that represent the goals, tasks, actions, or decisions that are relevant to support the technology [111]. Stakeholders with different backgrounds identify their problems with the current health care delivery via the scenarios and articulate their ideas about how to solve the problems. In addition to this, they define who the relevant stakeholders (key stakeholders with a vested interest) are who are affected by the problems and solutions. It is important that the opinions of all those involved be taken into account, as exclusion can cause a negative effect on future collaboration. To facilitate the discussion and subsequent reflections, scenarios can be used that present conceptual models and multiangle viewpoints (political, social, clinical, and behavioral) [9,23].

#### Value Specification

Consequently, *value specification* implies the recognition and quantification of the economic, medical, social, or behavioral values of the key stakeholders [58,110]. The most favorable solutions along with user and organizational requirements emerge from this process (user requirements, value drivers; see Figure 1). The value specification process elaborates on the outcomes of the contextual inquiry. In this cycle, the key stakeholders determine their values (economic, social, and behavioral) and rank them based on the importance of finding solutions for the identified problem(s).

The value-ranking method we use is based on multicriteria decision-making techniques (such as the analytic hierarchy/network process) that score attributes relatively and according to their hierarchy [112]. Value specification refers to goal setting and to defining the functional and organizational requirements to realize the values. It is aimed at exploring what health care improvements are foreseen and what the possibilities or expected limitations are to realize the values. The specified values have to be translated by the stakeholders into functionalities of the design and critical factors for the implementation. For example, during the process of developing a teledermatology application, the key stakeholders identified problems with measuring the possible risks of infection of diabetic feet and insufficient communication between caregivers (general practitioner and dermatologist). The values they formulated were higher quality of care and efficiency to reduce the number of errors and misinterpretations. The technology should therefore have functions to measure the conditions of the wound in an objective and standard way, and the measurements should be communicable in a standardized way. At the same time, the development team gains insights into how to shape the business to offer the values. For example: what are the costs and benefits of teledermatology for the general practitioner, specialist, and patients? The best solution to the problem, the one that emerges from ranking the obtained values with the stakeholders, is the one that will be most beneficial to, and favored by, the key stakeholders.

#### Design

This is followed by *design*, which refers to building prototypes that fit with the values and user requirements. The design cycle involves the translation of functional requirements into technical requirements and prototypes, given the specified values and goals of the eHealth project. The project management team has to visualize the translation into mock-ups keeping in mind the values, goals, and tasks that have to be fulfilled. Mock-ups, storyboards, or paper prototypes [101] are created and tested sequentially and iteratively with intended users [23,26,29,48,60] and, as a result, the prototypes are refined. The prototypes are tested in real-life situations. The intended users are invited in several rounds via concrete scenarios or tasks to give feedback and to test whether the prototypes match their expectations and mental models (way of thinking and working). To fine-tune the format and content, persuasive techniques [71,73] and card sorting [113,114] can be used to match the information to users’ needs. For example, to increase adherence to medical protocols, these documents can be made more user friendly via Web-based communication systems using card sorting to attune the information structure to their mental models and information-searching behavior [21]. In general, the quality of the design can be assessed at different levels [32]: *system quality*, creating technology that is user friendly, is easy to manage, and matches end users’ profiles and roles or tasks in the care-delivery process; *content quality*, creating information that is meaningful (accuracy, legibility, comprehensiveness, consistency, and reliability) and persuasive (format fits with users profile); and *service quality*, providing an e-service that is adequate (timely, responsive, and empathetic) and feasible, and measuring the degree to which the e-service is compatible with the health care system.

#### Operationalization

Operationalization concerns the actual introduction, adoption, and employment of the technology in practice. The cycle consists of enabling and reinforcing activities and mobilizing resources for training, education, and deployment of the eHealth technology in daily practice. Disregarding these conditions may limit the technology’s usefulness and delay decision making. An operationalization plan is needed to guide the adoption process—for example, regulations, opinion leaders, triggers, and incentives for using the eHealth technologies [115]—and to create momentum for managing the innovation [28,116]. A business case can be developed that consists of several scenarios, in-depth financial analyses, details about arrangements with other organizations, concrete plans for roles and activities, etc. The implementation of a prototype is discussed via filling up a *business model* canvas [58,106,107] with obtained critical factors, which allows discussion on how to form the business and what strategic choices must be made in order to implement the eHealth technology. A business model is to be developed to steer the adoption process—for example, with regard to internal and external incentives for using the eHealth technologies (the details of business modeling will be explained in a subsequent paper).

#### Summative Evaluation

Finally, *summative evaluation* refers to the actual uptake of a technology (its usage) and the assessment of the impact of eHealth technologies in terms of clinical, organizational, and behavioral terms. The summative evaluation measures the outcomes at different levels: the usage of a technology and the effects on performance criteria for high-quality care [28,117]. The critical factors that became apparent influence the uptake and impact of the eHealth technology and therefore need to be closely monitored. If certain critical factors start to have negative effects in the summative evaluation phase, the choice needs to be made to iterate to change and improve the current implementation or totally redesign the implementation. This way, the eHealth technology can be kept sustainable and cost effective.

## Conclusion

In this paper we have argued for a holistic approach for the development of eHealth technologies that integrates persuasive health technology theories with a managerial approach (business modeling) to improve the uptake and impact of eHealth technologies in practice. Based on reviews of current eHealth frameworks and on empirical research, we formulated principles for developing eHealth technologies. These principles are the bedrock of the holistic framework we introduced in this paper. The framework provides a comprehensive development strategy; it is suggested that, in the real world, time, policy, and financial considerations can hinder the use of the full framework. The framework is flexible and provides strategies that can be used in part and in a forward (development) and backward (summative evaluation) process. Deficiencies in these processes can be recognized and articulated to determine the bandwidth of innovations. The framework can serve as a debating instrument to clarify areas that would otherwise remain unanswered, unclear, or unknown.

The framework is currently being applied in several research projects. The preliminary results show the benefits of the holistic approach. Technology is not considered as a tool or end in itself, but as a catalyst for innovation. In a teledermatology project about wound care (see Multimedia Appendix 4), it became clear via contextual inquiry that stakeholders demanded a more comprehensive solution than just a tool for taking pictures of a wound. Via value creation, a new infrastructure for replacing hospital care with home-based care was developed with the aid of the roadmap. Via business modeling, the project management team had to think about how technology could improve the wound care process and what the implications were for replacing hospital care with home-based care from a socioeconomic and medical perspective. This resulted in a new infrastructure for teledermatology in health care and a business model that guided the deployment of the eHealth technology. The implications for reimbursement and medical practice were articulated before the production of eHealth technology, and as such the critical factors for deployment were translated into business models that could be discussed with key stakeholders to find out what model suited the eHealth technology best.

Stakeholder engagement resulted in commitment, trust, and a positive attitude toward investments in eHealth technologies (findings from the EurSafety Health-net project; see Multimedia Appendix 4). The participatory development of end users throughout the development process resulted in better adherence to technologies and fewer errors. Moreover, stakeholder involvement resulted in a rethinking of how technology can innovate health care. Standard care tends to be protocol driven; these protocols are often impossible to find or are inaccurate, or too rigid, to be manageable in practice. In the EurSafety Health-net project, medical staff were involved in the codesign of a new approach for antibiotic stewardship (to avoid resistance to infections). The value-creation process resulted in a reconsideration of the values of medical thinking and how technology can fit in with medical practice. This resulted in a shift from solely protocol-driven thinking to an infrastructure for the improved management of antibiotics via Web-based systems for communication and information.

In a companion paper in this journal, we elaborate on the business modeling aspects required to foster the sustainability of eHealth technologies [118]. To support a discussion about the development of eHealth technologies we created a Wiki to accompany our framework. The Wiki, available at http://ehealthwiki.org, is an open and collaborative approach to the development of eHealth technologies. It will provide an expanding and continuously evolving collection of instruments and tools to assist developers, researchers and policy makers. In a following paper we will describe the Wiki.

## Multimedia Appendix 1

In- and excluded eHealth journal papers.

## Multimedia Appendix 2

Target group, goal, foundation, definition of technology.

## Multimedia Appendix 3

Strategies and principles for evaluation, design, and implementation of eHts.

## Multimedia Appendix 4

Case studies.

## Abbreviations

fr.: framework
RCT: randomized controlled trial
