# Supplementary material for: A Holistic Framework to Improve the Uptake and Impact of eHealth Technologies
Source: J Med Internet Res. 2011 Dec 13;13(4):e111. doi: 10.2196/jmir.1672 (PMC3278097; doi:10.2196/jmir.1672)
Supplement: Supplementary file 1 [file jmir_v13i4e111_app1.pdf]

Multimedia Appendix 1. In- and excluded eHealth journal papers

Refs:	Category:	In- and exclusion criteria:
[1-16]	Framework	Included (16); papers describing strategies or principles for improving the development of eHts and presenting hands-on guidelines or quality criteria
[17-44]	Non-framework	Excluded (28); papers describing strategies or principles for improving the development of eHts without presenting a hands-on guideline or quality criteria
[45-60]	General	Excluded (16); papers not describing strategies or principles for improving the development of eHts

1. Esser P, Goossens R. A framework for the design of user-centred teleconsulting systems. J Telemed Telecare 2009;15(1):32-39. [PMID:19139218]
2. Catwell L, Sheikh A. Evaluating eHealth interventions: the need for continuous systemic evaluation. PLoS Med 2009;6(8):e1000126. [PMID:19688038]
3. Yusof MM, Kuljis J, Papazafeiropoulou A, Stergioulas LK. An evaluation framework for health information systems: human, organization and technology-fit factors (HOT-fit). Int J Med Inform 2008;77(6):386-398. [PMID:17964851]
4. Hamid A, Sarmad A. Evaluation of e-health services: user's perspective criteria. Transforming government: people, process and policy 2008;2(4):243-255. [DOI:10.1108/17506160810917945]
5. Pagliari C. Design and evaluation in eHealth: challenges and implications for an interdisciplinary field. J Med Internet Res 2007;9(2):e15. [PMID:17537718]
6. Kaufman D, Roberts WD, Merrill J, Lai TY, Bakken S. Applying an evaluation framework for health information system design, development, and implementation. Nurs Res 2006;55(2 Suppl):S37-42. [PMID:16601633]
7. Dansky K, Thompson D, Sanner T. A framework for evaluating eHealth research. Eval Program Plann 2006;29(4):397-404. [PMID:17950868]
8. Van der Meijden MJ, Tange HJ, Troost J, Hasman A. Determinants of success of inpatient clinical information systems: a literature review. J Am Med Inform Assoc May-Jun 2003;10(3):235-243. [PMID:12626373]
9. Shaw NT. CHEATS: a generic information communication technology (ICT) evaluation framework. Comput Biol Med 2002;32(3):209-220. [PMID:11922936]
10. Kazanjian A, Green CJ. Beyond effectiveness: the evaluation of information systems using A Comprehensive Health Technology Assessment Framework. Comput Biol Med 2002;32(3):165-177. [PMID:11922933]
11. Kushniruk A. Evaluation in the design of health information systems: application of approaches emerging from usability engineering. Comput Biol Med May 2002;32(3):141-149. [PMID:11922931]
12. Hebert M. Telehealth success: evaluation framework development. Stud Health Technol Inform 2001;84(Pt 2):1145-1149. [PMID:11604908]
13. Eysenbach G. A framework for evaluating e-health: systematic review of studies assessing the quality of health information and services for patients on the Internet. J Med Internet Res 2000;2(suppl2) [DOI:10.2196/jmir.2.suppl2.e13]
14. Eng TR, Gustafson DH, Henderson J, Jimison H, Patrick K. Introduction to evaluation of interactive health communication applications. Science Panel on Interactive Communication and Health. Am J Prev Med Jan 1999;16(1):10-15. [PMID:9894549]
15. Jai Ganesh A. E-health - drivers, applications, challenges ahead and strategies: a conceptual framework. Ind J Med Inform 2004;1:39-47.
16. Kukafka R, Johnson SB, Linfante A, Allegrante JP. Grounding a new information technology implementation framework in behavioral science: a systematic analysis of the literature on IT use. J Biomed Inform 2003;36(3):218-227. [PMID:14615230]

17. Resnicow K, Strecher V, Couper M, et al. Methodologic and design issues in patient-centered e-health research. Am J Prev Med Jan 2010;38(1):98-102. [PMID:20117564]
18. Atienza AA, Hesse BW, Gustafson DH, Croyle RT. E-health research and patient-centered care examining theory, methods, and application. Am J Prev Med Jan 2010;38(1):85-88. [PMID:20117562]
19. Lilford RJ, Foster J, Pringle M. Evaluating eHealth: how to make evaluation more methodologically robust. PLoS Med 2009;6(11):e1000186. [PMID:19956674]
20. Bates DW, Wright A. Evaluating eHealth: undertaking robust international cross-cultural eHealth research. PLoS Med 2009;6(9):e1000105. [PMID:19753106]
21. Yusof MM, Papazafeiropoulou A, Paul RJ, Stergioulas LK. Investigating evaluation frameworks for health information systems. Int J Med Inform Jun 2008;77(6):377-385. [PMID:17904898]
22. Ovretveit J, Scott T, Rundall TG, Shortell SM, Brommels M. Improving quality through effective implementation of information technology in healthcare. Int J Qual Health Care 2007;19(5):259-266. [PMID:17717038]
23. Hesse BW, Shneiderman B. eHealth research from the user's perspective. Am J Prev Med 2007;32(5 Suppl):S97-103. [PMID:17466825]
24. Glasgow RE. eHealth evaluation and dissemination research. Am J Prev Med May 2007;32(5 Suppl):S119-126. [PMID:17466816]
25. Curry SJ. eHealth research and healthcare delivery beyond intervention effectiveness. Am J Prev Med May 2007;32(5 Suppl):S127-130. [PMID:17466817]
26. Chiasson M, Reddy M, Kaplan B, Davidson E. Expanding multi-disciplinary approaches to healthcare information technologies: what does information systems offer medical informatics? Int J Med Inform Jun 2007;76 Suppl 1:S89-97. [PMID:16769245]
27. Broens TH, Huis in't Veld RM, Vollenbroek-Hutten MM, Hermens HJ, van Halteren AT, Nieuwenhuis LJ. Determinants of successful telemedicine implementations: a literature study. J Telemed Telecare 2007;13(6):303-309. [PMID:17785027]
28. Atienza AA, Hesse BW, Baker TB, et al. Critical issues in eHealth research. Am J Prev Med 2007;32(5):S71-S74. [PMID:17466821]
29. Ahern D. Challenges and opportunities of eHealth research. Am J Prev Med 2007;32(5):S75-S82. [PMID:17466822]
30. Rigby M. Essential prerequisites to the safe and effective widespread roll-out of e-working in healthcare. Int J Med Inform 2006;75(2):138-147. [PMID:16085451]
31. Kern J. Evaluation of teleconsultation systems. Int J Med Inf 2006;75(3-4):330-334. [PMID:16143564]
32. Doolittle GC, Spaulding RJ. Defining the needs of a telemedicine service. J Telemed Telecare 2006;12(6):276-284. [PMID: 17022834]
33. Taylor P. Evaluating telemedicine systems and services. J Telemed Telecare 2005;11(4):167-177. [DOI:10.1258/1357633054068955]
34. Grigsby J, Brega AG, Devore PA. The evaluation of telemedicine and health services research. Telemed J E Health Jun 2005;11(3):317-328. [PMID:16035929]
35. Gagnon MP, Scott RE. Striving for evidence in e-health evaluation: lessons from health technology assessment. J Telemed Telecare 2005;11 Suppl 2:S34-36. [PMID:16375790]
36. Currie LM. Evaluation frameworks for nursing informatics. Int J Med Inform Dec 2005;74(11-12):908-916. [PMID:16099711]
37. Gustafson DH, Wyatt JC. Evaluation of ehealth systems and services. BMJ 2004;328(7449):1150. [PMID:15142895]
38. May C, Harrison R, Finch T, MacFarlane A, Mair F, Wallace P. Understanding the normalization of telemedicine services through qualitative evaluation. J Am Med Inform Assoc 2003;10(6):596-604. [PMID:12925553]
39. Ammenwerth E, Graber S, Herrmann G, Burkle T, Konig J. Evaluation of health information systems-problems and challenges. Int J Med Inform Sep 2003;71(2-3):125-135. [PMID:14519405]
40. Eng TR. eHealth research and evaluation: challenges and opportunities. J Health Commun 2002;7(4):267-272. [PMID:12356287]

41. Wootton R, Hebert MA. What constitutes success in telehealth? J Telemed Telecare 2001;7 Suppl 2:3-7. [PMID:11546704]
42. Kaplan B. Evaluating informatics applications - clinical decision support systems literature review. Int J Med Inform 2001;64(1):15-37. [PMID:11673100]
43. Kaplan B. Evaluating informatics applications - some alternative approaches: theory, social interactionism, and call for methodological pluralism. Int J Med Inform Nov 2001;64(1):39-56. [PMID:11673101]
44. Stead WW, Haynes RB, Fuller S, et al. Designing medical informatics research and library--resource projects to increase what is learned. J Am Med Inform Assoc Jan-Feb 1994;1(1):28-33. [PMID:7719785]
45. Eysenbach G. Poverty, human development, and the role of eHealth. J Med Internet Res 2007;9(4):e34. [PMID:17951217]
46. Rheuban KS. The role of telemedicine in fostering health-care innovations to address problems of access, specialty shortages and changing patient care needs. J Telemed Telecare 2006;12 Suppl 2:S45-50. [PMID:16989674]
47. Griffiths F, Lindenmeyer A, Powell J, Lowe P, Thorogood M. Why are health care interventions delivered over the internet? A systematic review of the published literature. J Med Internet Res 2006;8(2):e10. [PMID:16867965]
48. Ahern D, Kreslake J, Phalen J. What is eHealth (6): perspectives on the evolution of eHealth research. J Med Internet Res 2006;8(1):e4. [PMID:16585029]
49. Wyatt JC, Sullivan F. eHealth and the future: promise or peril? BMJ Dec 10 2005;331(7529):1391-1393. [PMID:16339252]
50. Pagliari C, Sloan D, Gregor P, et al. What is eHealth (4): a scoping exercise to map the field. J Med Internet Res 2005;7(1):e9. [PMID:15829481]
51. Oh H, Rizo C, Enkin M, Jadad A. What is eHealth (3): a systematic review of published definitions. J Med Internet Res 2005;7(1):e1. [PMID:15829471]
52. Jones R, Rogers R, Roberts J, et al. What is eHealth (5): a research agenda for eHealth through stakeholder consultation and policy context review. J Med Internet Res 2005;7(5):e54. [PMID:16403718]
53. Hjelm NM. Benefits and drawbacks of telemedicine. J Telemed Telecare 2005;11(2):60-70. [PMID:15829049]
54. Demiris G, Tao D. An analysis of the specialized literature in the field of telemedicine. J Telemed Telecare 2005;11(6):316-319. [PMID:16168169]
55. Rodrigues R. Opportunities and challenges in the deployment of global e-health. Int J Healthcare Technology and Management 2003;5(3/4/5):335-357. [DOI:10.1504/IJHTM.2003.004173]
56. Bodenheimer T, Grumbach K. Electronic technology: a spark to revitalize primary care? JAMA Jul 9 2003;290(2):259-264. [PMID:12851283]
57. Wootton R. Recent advances: telemedicine. BMJ Sep 8 2001;323(7312):557-560.
58. Eysenbach G. What is e-health? J Med Internet Res Apr-Jun 2001;3(2):e20. [PMID:11720962]
59. Della Mea V. What is e-health (2): the death of telemedicine? J Med Internet Res 2001;3(2):E22. [PMID:11720964]
60. Kassirer JP. Patients, physicians, and the Internet. Health Aff (Millwood) Nov-Dec 2000;19(6):115-123. [PMID:11192394]

Note. The framework numbers 1-16 correspond as follows to the reference numbers of the manuscript:

Framework	Reference	Corresponding author
fr.1	[104]	Esser & Goossens
fr.2	[23]	Catwell & Sheikh
fr.3	[28]	Yusof et al.
fr.4	[50]	Hamid & Sarmad
fr.5	[48]	Pagliari
fr.6	[29]	Kaufman et al.
fr.7	[6]	Dansky et al.

fr.8	[30]	Van der Meijden et al.
fr.9	[27]	Shaw
fr.10	[49]	Kazanjian & Green
fr.11	[60]	Kushniruk
fr.12	[33]	Hebert
fr.13	[117]	Eysenbach
fr.14	[51]	Eng et al.
fr.15	[52]	Jai Ganesh
fr.16	[26]	Kukafka et al.
