# Supplementary material for: A Holistic Framework to Improve the Uptake and Impact of eHealth Technologies
Source: J Med Internet Res. 2011 Dec 13;13(4):e111. doi: 10.2196/jmir.1672 (PMC3278097; doi:10.2196/jmir.1672)
Supplement: Supplementary file 2 [file jmir_v13i4e111_app2.pdf]

Multimedia Appendix 2. Target group, goal, foundation, definition of technology

fr.	Author, year, title	Target group, goal, foundation, and definition of eHealth and focus on technology
1	Esser et al., 2009,<br>A framework for the design of user-centred teleconsulting systems [1]	<u>Target group</u><br>Design professionals (not specified)<br><br><u>Goal</u><br>The framework aims to improve the design process by helping the design professional to obtain a quick overview of all the aspects within the context that are relevant to the users of a teleconsultation system to obtain improved health, satisfaction, and ultimately acceptance. The authors state that the framework and the checklist are especially set up for, and thus restricted to, the context of patient-provider teleconsultation.<br><br><u>Foundation</u><br>(1) Doctor-patient communication models, eg, Miller (2002) [17], and Roter et al. (1988) [18]<br>(2) Technology acceptance models, eg, Unified Theory of Acceptance and Use of Technology (UTAUT) [19-20]<br>(3) Technology-mediated communication theory, eg, Media Richness Theory [21]<br>Two field experts validated the relevance of the framework.<br><br><u>Definition of technology</u><br>Telemedicine; own definition:<br>*"Information and communication technologies for the exchange of medical information and expertise in the delivery of clinical services to patients, i.e. telemedicine."*<br>Examples mentioned:<br>- telemedicine<br>- teleconsultations
2	Catwell & Sheikh, 2009,<br>Evaluating eHealth interventions: the need for continuous systemic evaluation [2]	<u>Target group</u><br>Designers (not specified)<br><br><u>Goal</u><br>The overall aim of this model is to maximize the benefits while minimizing any risks associated with the eHealth intervention. This model has the additional advantage of providing a means to understand the implementation process.<br><br><u>Foundation</u><br>Literature:<br>- cognitive and usability engineering methods for the evaluation of clinical information systems (Kushniruk & Patel, 2004 [22])<br>- sociotechnical and contextual considerations (Black et al., 2008 [23])

		<u>Definition of technology</u><br>eHealth; definition based on Eysenbach (2001) [24]:<br>*"…an emerging field of medical informatics, referring to the organization and delivery of health services and information using the Internet and related technologies. In a broader sense, the term characterizes not only a technical development, but also a new way of working, an attitude, and a commitment for networked, global thinking, to improve healthcare locally, regionally and worldwide by using information and communication technology."*<br>Examples mentioned:<br>- telemedicine interventions: telemonitoring devices (eg, teleradiology, telecardiology, teleconsulting, telesurgery).<br>- electronic medical (health) records<br>- health portals (eg, Google Health)
3	Yusof et al., 2008,<br>An evaluation framework for health information systems: human, organization and technology-fit factors [3]	<u>Target group</u><br>Researchers and practitioners (clinicians/GPs)<br><br><u>Goal</u><br>The framework aims to assist researchers and practitioners to unfold and understand the perceived complexity of health information system evaluation (on the performance, effectiveness, and impact of HIS).<br><br><u>Foundation</u><br>(1) The IS Success Model of DeLone & McLean [25-26]<br>(2) The IT-Organization Fit Model adapted from Scott Morton [27]<br>(3) Literature review; critical appraisal of health information systems studies<br>(4) Pilot testing developed framework (case study clinical setting)<br><br><u>Definition of technology</u><br>Health Information Systems (HIS); not defined<br>Examples mentioned:<br>- computing and telecommunications devices: digital fundus imaging system for diabetic retinopathy<br>- electronic patient records
4	Hamid & Sarmad, 2008,<br>Evaluation of e-health services: user's perspective criteria [4]	<u>Target group</u><br>Not mentioned<br><br><u>Goal</u><br>The framework aims to influence users' utilization and satisfaction of eHealth services.<br><br><u>Foundation</u><br>(1) behavioral theories, eg, technology acceptance (TAM) [28], and diffusion of innovations (DOI) [29]<br>(2) broad examination of existing evaluation initiatives based on eHealth services case studies<br>The authors argue that an eHealth services evaluation framework

		should be criteria-based, while the criteria can be grounded in, and derived from, one or more specific perspectives or theories, and cannot be entirely framed within the bounds of a single theory or perspective. Understanding the *multi-disciplinary nature of eHealth services evaluation* and the challenges that it faces is the first requisite towards dealing effectively with the complexities and overcoming the barriers of eHealth services evaluation.<br><br>Definition of technology<br>eHealth; definition by the WHO/Canada's Health Informatics Association (see Oh et al., 2005 [30]):<br>*"the leveraging of the information and communication technology to connect provider and patients and governments; to educate and inform healthcare professionals, managers and consumers; to stimulate innovation in care delivery and health system management; and, to improve our healthcare system."*<br>Examples mentioned: none
5	Pagliari, 2007,<br>Design & evaluation in eHealth: challenges and implications for an interdisciplinary field [5]	Target group<br>Software developers and health services researchers<br><br>Goal<br>The aim of the framework is to improve the quality and effectiveness of eHealth.<br><br>Foundation<br>(1) Software engineering & System Development Life Cycle Models (SDLC), eg, Waterfall [31], Spiral [32], Star Model [33]<br>(2) Health service research evaluation methods [34-36]<br>(3) Non-systematic review of literature interdisciplinary methods in medical informatics<br><br>Definition of technology<br>eHealth; own definition:<br>*"For the purposes of this paper, the term* eHealth *is used broadly as a synonym for health informatics or medical informatics and health services research for health technology assessment and health systems research."*<br>Examples mentioned: none
6	Kaufman et al., 2006,<br>Evaluation framework for health information system design, development and implementation [6]	Target group<br>Researchers and designers<br><br>Goal<br>The framework aims:<br>(1) To enhance the potential of eHts to influence the healthcare process positively.<br>(2) To provide coherence and structure to research in informatics.<br>(3) To help researchers focus on the types of evaluation objectives to be accomplished in all phases of system development.<br><br>Foundation

		Based on the evaluation framework of Stead et al. (1994) [37]; the central premise of the Stead et al. framework is the importance of a continuous, systematic, and rigorous approach to evaluation.<br><br><u>Definition of technology</u><br>Health Information Systems (HIS); not defined<br>Examples mentioned: none
7	Dansky et al., 2006,<br>A framework for evaluating eHealth research [7]	<u>Target group</u><br>Researchers and others (not specified)<br><br><u>Goal</u><br>The framework aims:<br>(1) To overcome the challenges of implementing eHealth programs.<br>(2) To assist eHealth researchers and others to build and evaluate effective eHealth programs.<br><br><u>Foundation</u><br>Health Insurance Portability and Accountability Act (regulations).<br><br><u>Definition of technology</u><br>eHealth; definition by Eng (2002) [38]:<br>*“The term ‘eHealth’ has emerged as a central, unifying definition of multiple technologies and modalities; essentially, it refers to: the use of emerging information and communication technology, especially the Internet, to improve or enable health and health care”*<br>Examples mentioned:<br>- telemedicine/telehealth for data transmission: one-way systems (eg, teleradiology), store-and-forward systems (eg, teleconsultation), complex video interactions with medical devices. Example: home health services for patients with chronic illness (eg, diabetes wound care; chronic congestive heart failure monitoring)<br>- wireless technologies: electronic medical records (web portals), personal digital assistants used by physicians<br>- online chat (individually or in virtual communities)<br>- health information and services on the web (web-based programs)
8	Van der Meijden et al., 2003,<br>Determinants of success of inpatient clinical information systems: a literature review [8]	<u>Target group</u><br>Not mentioned<br><br><u>Goal</u><br>The aim of the framework is to foster the success of clinical information systems.<br><br><u>Foundation</u><br>- DeLone & McLean’s Dimensions of IS Success [30,31]<br>- review of literature on patient care information systems<br><br><u>Definition of technology</u><br>Health Information Systems (HIS); own definition:

		"A patient care information system was defined as a clinical information system in use in inpatient settings, requiring data entry and data retrieval by health care professionals themselves."<br>Examples mentioned:<br>- general systems: hospital information systems, nursing (bedside) documentation systems, computerized medical record systems, physician order entry systems<br>- specific systems: intensive care unit systems, automated anaesthesia record-keeping systems
9	Shaw, 2002, 'CHEATS': a generic information communication technology (ICT) evaluation framework [9]	<u>Target group</u><br>Not mentioned<br><br><u>Goal</u><br>Not mentioned<br><br><u>Foundation</u><br>Empirical evidence in multiple clinical settings<br>Multiple clinical settings:<br>- telepsychiatry<br>- teledermatology<br>- tele-education<br><br><u>Definition of technology</u><br>Information Communication Technology (ICT); not defined<br>Examples mentioned:<br>- GP clinical information systems<br>- electronic discharge summaries<br>- online information services<br>- telemedicine systems
10	Kazanjian & Green, 2002, Beyond effectiveness: the evaluation of information systems using a comprehensive health technology assessment framework [10]	<u>Target group</u><br>Decision-makers (policy-makers, administrative developers of information systems)<br><br><u>Goal</u><br>The aim of the framework is to provide an empirical, evidence based foundation for health technology decisions.<br><br><u>Foundation</u><br>- theories of epidemiology, sociology, economics, system science<br>- critical theory to healthcare evaluation<br>- Health Technology Assessment (HTA) [39]; HTA is the systematic evaluation and synthesis of evidence on the properties, effects, and other impacts of health technologies<br><br><u>Definition of technology</u><br>Health Information Systems (HIS); definition by the Institute of Medicine (2001):<br>*"Health information systems can be classified as health technologies, which - together with devices, drugs, and medical or surgical procedures -include the 'organizational=administrative*

		and support systems within which health care is delivered.”<br>Examples mentioned:<br>- telehealth application: consultations for populations with restricted access
11	Kushniruk, 2002, Evaluation in the design of health information systems: application of approaches emerging from usability engineering [11]	<u>Target group</u><br>Not mentioned<br><br><u>Goal</u><br>Not mentioned<br><br><u>Foundation</u><br>- System Development Life Cycle (SDLC) models [31-33]<br>- usability engineering model (rapid iterative software development)<br><br><u>Definition of technology</u><br>Health Information Systems (HIS); not defined<br>Examples mentioned:<br>- web-based patient record systems<br>- decision support tools<br>- educational tools
12	Hebert, 2001, Telehealth success: evaluation framework development [12]	<u>Target group</u><br>Not mentioned<br><br><u>Goal</u><br>The framework aims:<br>(1) To develop a body of knowledge around telehealth evaluations and supporting more advanced research efforts.<br>(2) To guide eHealth investments about where telehealth is effective as well as what variables demonstrate telehealth success (eg, quality patient care, user satisfaction).<br><br><u>Foundation</u><br>- Donabedian’s model for assessing the quality of care (1980) [40]<br>- DeLone & McLean’s Dimensions of IS Success (1992) [25]<br>- Health Technology Assessment (HTA) [39]<br>The evaluation framework is being tested through mapping of project reports identified in a literature review. Quasi-experimental studies in tele-homecare are expected to report their findings using this framework.<br><br><u>Definition of technology</u><br>Telehealth; definition by Field (1996) [41] and Reid (1996) [42]:<br>*“The term “telehealth” is used to describe the exchange of health information and provide health care services through electronic information and communication technology (ICT), where participants are separated by geographic, time, social and cultural barriers.”*<br>Examples mentioned:<br>- clinical application (eg, telepsychiatry, teleradiology)

		- characteristics of information being transmitted (eg, audio, visual, text, data)<br>- temporal relationships (eg, synchronous, real-time, asynchronous, store and forward).
13	Eysenbach, 2000,<br>A framework for evaluating eHealth: systematic review of studies assessing the quality of health information and services for patients on the Internet [13]	<u>Target group</u><br>Not mentioned<br><br><u>Goal</u><br>Conceptual and methodological framework for describing, comparing, and analyzing the structure and quality of eHealth.<br><br><u>Foundation</u><br>- Donabedian's model for assessing the quality of care (1980) [40]<br>- systematic reviews assessing the quality of health information and services for patients on the internet<br><br><u>Definition of technology</u><br>eHealth; not defined<br>Examples mentioned:<br>- information on websites (eg, drug information on e-commerce sites)<br>- online consultations (eg, cyberdocs)<br>- communities: messages on mailing lists or Usenet newsgroups
14	Eng et al., 1999,<br>Evaluation framework for interactive health communication applications [14]	<u>Target group</u><br>Not mentioned<br><br><u>Goal</u><br>The framework aims to improve the quality and effectiveness of eHts.<br><br><u>Foundation</u><br>Making health communication programs work (National Cancer Institute, 1989 [43])<br><br><u>Definition of technology</u><br>Interactive Health Communication (IHC) applications; defined by Robinson et al. (1998) [44]:<br>*"the interaction of an individual (consumer, patient, caregiver, or professional with of through an electronic device or communication technology to access or transmit health information or receive guidance and support on health-related issues".*<br>Examples mentioned:<br>the authors use the term to refer to operational software programs or modules that interface with the end user; this includes:<br>- health information and support websites<br>- clinical decision-support and risk assessment software<br>According to the authors the term does <u>not</u> include applications that focus exclusively on administrative, financial, or clinical data (no health communication functions), such as:<br>- electronic medical records

		- dedicated clinical telemedicine applications<br>- expert clinical decision-support systems for providers
15	Jai Ganesh, 2004, eHealth - drivers, applications, challenges ahead and strategies: a conceptual framework [15]	<u>Target group</u><br>Not mentioned<br><br><u>Goal</u><br>The framework aims to foster the widespread adoption of eHts and successful deliverance of eHts.<br><br><u>Foundation</u><br>-WHO strategy to design, reconfigure healthcare systems to better meet the needs of people with chronic illnesses: Innovative care for chronic conditions: building blocks for action [45].<br>- Doolittle and Cook's needs assessment model (2006) [46]<br><br><u>Definition of technology</u><br>eHealth; own definition:<br>*"eHealth refers to any use of an electronic information and communication technology to promote health or improve health care."*<br>According to the author, eHealth applications can be grouped under: consumer health, clinical care, financial/administrative transactions, public health, professional education, and biomedical research. The infrastructure of an eHealth program consists of 3 components: human, technical, and medical.<br>Examples mentioned:<br>- websites addressing consumer health needs<br>- telecommunications<br>- electronic medical records<br>- home care technologies<br>- telemedicine<br>- clinical transactions systems<br>- clinical decision support systems<br>- health diagnostic equipments
16	Kukafka et al., 2003, Grounding a new information technology implementation framework in behavioral science: a systematic analysis of the literature on IT use [16]	<u>Target group</u><br>Health planners, developers (not specified)<br><br><u>Goal</u><br>The framework is intended to guide synthesis of more than one theoretical perspective for the purpose of planning multi-level interventions to enhance IT use.<br><br><u>Foundation</u><br>- systematic literature review on IT use (behavioral theories and models that explain IT usage); the authors undertook a systematic literature analysis to confirm their assertion that the literature on IT use behavior does not include a multi-level approach.<br>- The integrative framework is adapted from PRECEDE/PROCEDE (Green and Kreuter, 1999 [47]), a conceptual framework used by health planners.

		Definition of technology<br>Information Technology (IT) in healthcare; not defined<br>Examples mentioned:<br>- electronic medical record

References

1. Esser P, Goossens R. A framework for the design of user-centred teleconsulting systems. J Telemed Telecare 2009;15(1):32-39. [PMID:19139218]
2. Catwell L, Sheikh A. Evaluating eHealth interventions: the need for continuous systemic evaluation. PLoS Med 2009;6(8):e1000126. [PMID:19688038]
3. Yusof MM, Kuljis J, Papazafeiropoulou A, Stergioulas LK. An evaluation framework for health information systems: human, organization and technology-fit factors (HOT-fit). Int J Med Inform 2008;77(6):386-398. [PMID:17964851]
4. Hamid A, Sarmad A. Evaluation of e-health services: user's perspective criteria. Transforming government: people, process and policy 2008;2(4):243-255. [DOI:10.1108/17506160810917945]
5. Pagliari C. Design and evaluation in eHealth: challenges and implications for an interdisciplinary field. J Med Internet Res 2007;9(2):e15. [PMID:17537718]
6. Kaufman D, Roberts WD, Merrill J, Lai TY, Bakken S. Applying an evaluation framework for health information system design, development, and implementation. Nurs Res 2006;55(2 Suppl):S37-42. [PMID:16601633]
7. Dansky K, Thompson D, Sanner T. A framework for evaluating eHealth research. Eval Program Plann 2006;29(4):397-404. [PMID:17950868]
8. Van der Meijden MJ, Tange HJ, Troost J, Hasman A. Determinants of success of inpatient clinical information systems: a literature review. J Am Med Inform Assoc May-Jun 2003;10(3):235-243. [PMID:12626373]
9. Shaw NT. CHEATS: a generic information communication technology (ICT) evaluation framework. Comput Biol Med 2002;32(3):209-220. [PMID:11922936]
10. Kazanjian A, Green CJ. Beyond effectiveness: the evaluation of information systems using A Comprehensive Health Technology Assessment Framework. Comput Biol Med 2002;32(3):165-177. [PMID:11922933]
11. Kushniruk A. Evaluation in the design of health information systems: application of approaches emerging from usability engineering. Comput Biol Med May 2002;32(3):141-149. [PMID:11922931]
12. Hebert M. Telehealth success: evaluation framework development. Stud Health Technol Inform 2001;84(Pt 2):1145-1149. [PMID:11604908]
13. Eysenbach G. A framework for evaluating e-health: systematic review of studies assessing the quality of health information and services for patients on the Internet. J Med Internet Res 2000;2(suppl2) [DOI:10.2196/jmir.2.suppl2.e13]
14. Eng TR, Gustafson DH, Henderson J, Jimison H, Patrick K. Introduction to evaluation of interactive health communication applications. Science Panel on Interactive Communication and Health. Am J Prev Med Jan 1999;16(1):10-15. [PMID:9894549]
15. Jai Ganesh A. E-health - drivers, applications, challenges ahead and strategies: a conceptual framework. Ind J Med Inform 2004;1:39-47.
16. Kukafka R, Johnson SB, Linfante A, Allegrante JP. Grounding a new information technology implementation framework in behavioral science: a systematic analysis of the literature on IT use. J Biomed Inform 2003;36(3):218-227. [PMID:14615230]

17. Miller A. Telemedicine and doctor-patient communication: a theoretical framework for evaluation. J Telemed Telecare 2002;8(6):311-318. [PMID:12537917]
18. Roter DL, Hall JA, Katz NR. Patient-physician communication: a descriptive summary of the literature. Patient Educ Couns 1988;12(2):99-119. [DOI:10.1016/0738-3991(88)90057-2]
19. Chang I. Physicians' acceptance of pharmacokinetics-based clinical decision support systems. Expert Syst Appl 2007;33(2):296-303. [DOI:10.1016/j.eswa.2006.05.001]
20. Venkatesh V, Morris MG, Davis GB, Davis FD. User acceptance of information technology: toward a unified view. Mis Q 2003;27(3):425-478. [DOI:10.2307/30036540]
21. Suh KS. Impact of communication medium on task performance and satisfaction: an examination of media-richness theory. Info Manage 1999;35(5):295-312. [DOI:10.1016/S0378-7206(98)00097-4]
22. Kushniruk AW, Patel VL. Cognitive and usability engineering methods for the evaluation of clinical information systems. J Biomed Inform Feb 2004;37(1):56-76. [PMID:15016386]
23. Black AD, Car J, Pagliari C, et al. The impact of eHealth on the quality and safety of health care: a systematic overview. PLoS Med 2011;8(1):e1000387. [PMID:21267058]
24. Eysenbach G. What is e-health? J Med Internet Res Apr-Jun 2001;3(2):e20. [PMID:11720962]
25. DeLone W, McLean E. Information systems success: the quest for the dependent variable. Inf Syst Res 1992;3(1):60-95. [DOI:10.1287/isre.3.1.60]
26. DeLone W, McLean E. The DeLone and McLean model of information systems success: a ten-year update. J Manage Inform Syst 2003;19(4):9-30. [ISSN:07421222]
27. Scott Morton MS. The corporation of the 1990's: information technology and organizational transformation. New York: Oxford University Press, 1991. [ISBN:978-0195063585]
28. Davis FD. Perceived usefulness, perceived ease of use, and user acceptance of information technology. Mis Q 1989;13(3):319-340. [DOI:10.2307/249008]
29. Rogers EM. Diffusion of innovation. New York: Free Press, 2003. [ISBN:978-0743222099]
30. Oh H, Rizo C, Enkin M, Jadad A. What is eHealth (3): a systematic review of published definitions. J Med Internet Res 2005;7(1):e1. [PMID:15829471]
31. Royce WW. Managing the development of large software systems: concepts and techniques. Proceedings of the 9th international conference on Software Engineering; 1987 Mar 30 - Apr 2; Los Alamitos, CA, USA. [ISBN:0897912160]
32. Boehm BW. A spiral model of software development and enhancement. Computer 1988;21(5):61-72. [DOI:10.1109/2.59]
33. Hartson HR, Hix D. Toward empirically derived methodologies and tools for human-computer interface development. International Journal of Man-Machine Studies 1989;31(4):477-494. [DOI:10.1016/0020-7373(89)90005-9]
34. Campbell NC, Murray E, Darbyshire J, et al. Designing and evaluating complex interventions to improve health care. BMJ 2007;334(7591):455-459. [PMID:17332585]
35. Zuber-Skerrit O. Models for action research. In: Pinchen S, Passfield R, editors. Moving on: creative applications of action learning and action research Queensland, Australia: Action Research, Action Learning and Process Management (ALARPM) Association; 1995:3-29. [ISBN:9780646259307]
36. Deming WE. Out of the crisis. Cambridge, MA: The MIT Press, 2000. [ISBN:0911379010]
37. Stead WW, Haynes RB, Fuller S, et al. Designing medical informatics research and library--resource projects to increase what is learned. J Am Med Inform Assoc Jan-Feb 1994;1(1):28-33. [PMID:7719785]
38. Eng TR. eHealth research and evaluation: challenges and opportunities. J Health Commun 2002;7(4):267-272. [PMID:12356287]
39. Jennett B. Health technology assessment. BMJ 1992;305(6845):67-68. [PMID:1638246]
40. Donabedian A. Explorations in quality assessment and monitoring. Volume 1: the definition of quality and approaches to its assessment. Ann Arbor, Michigan: Health Administration Press, 1980. [ISBN:978-0914904472]

41. Field ME. Telemedicine: a guide to assessing telecommunications for health care. Washington, DC: National Academy Press, 1996. [ISBN:9780309055314]
42. Reid JA. Telemedicine primer: understanding the issues. Billings, Montana: Artcraft Printers, 1996. [ISBN:9780965304504]
43. National Cancer Institute. Making health communication programs work. Bethesda, MD: US Department of Health and Human Services, 1989.
44. Robinson TN, Patrick K, Eng TR, Gustafson D. An evidence-based approach to interactive health communication: a challenge to medicine in the information age. Science Panel on Interactive Communication and Health. JAMA Oct 14 1998;280(14):1264-1269. [PMID:9786378]
45. World Health Organization. Innovative care for chronic conditions: building blocks for action. Geneva, Switserland: WHO Press, 2002. [ISBN:9241590173]
46. Doolittle GC, Spaulding RJ. Defining the needs of a telemedicine service. J Telemed Telecare 2006;12(6):276-284. [PMID: 17022834]
47. Task Force on Community Preventive Services. Recommendations for healthcare system and self-management education interventions to reduce morbidity and mortality from diabetes. Am J Prev Med May 2002;22(4 Suppl):10-14. [PMID:11985932]

Note. The framework numbers 1-16 correspond as follows to the reference numbers of the manuscript:

Framework	Reference	Corresponding author
fr.1	[104]	Esser & Goossens
fr.2	[23]	Catwell & Sheikh
fr.3	[28]	Yusof et al.
fr.4	[50]	Hamid & Sarmad
fr.5	[48]	Pagliari
fr.6	[29]	Kaufman et al.
fr.7	[6]	Dansky et al.
fr.8	[30]	Van der Meijden et al.
fr.9	[27]	Shaw
fr.10	[49]	Kazanjian & Green
fr.11	[60]	Kushniruk
fr.12	[33]	Hebert
fr.13	[117]	Eysenbach
fr.14	[51]	Eng et al.
fr.15	[52]	Jai Ganesh
fr.16	[26]	Kukafka et al.
