# Supplementary material for: A Holistic Framework to Improve the Uptake and Impact of eHealth Technologies
Source: J Med Internet Res. 2011 Dec 13;13(4):e111. doi: 10.2196/jmir.1672 (PMC3278097; doi:10.2196/jmir.1672)
Supplement: Supplementary file 3 [file jmir_v13i4e111_app3.pdf]

Multimedia Appendix 3. Strategies and principles for eHealth Research & Development

fr.	Author, year, title	Strategies and principles
1	Esser et al., 2009,<br>A framework for the design of user-centred teleconsulting systems [1]	The framework proposes a **user-centered design approach** for telemedicine systems by taking the first step of mapping the underlying **theoretical dimensions relevant for teleconsultations**, taking the patient-provider interaction as the starting point. User-centered design is a design approach in which the needs and requirements of users are considered at each stage of the design process.<br><br><u>User-centered design approach</u><br>Users: patients and healthcare providers<br><br><u>Theoretical dimensions relevant for teleconsultations</u><br>(1) individual context (patient, provider, disease characteristics)<br>(2) organizational context (eg, compatibility, facilitating conditions)<br>(3) technological context (eg, medium characteristics, mode/interactivity)<br>(4) teleconsultation process: communications & perceptions<br>(5) process evaluation: health outcomes, satisfaction, adoption
2	Catwell & Sheikh, 2009,<br>Evaluating eHealth interventions: the need for continuous systemic evaluation [2]	The framework proposes a comprehensive overall evaluation approach, one that encourages a **multifaceted, multidisciplined approach** and facilitates **continuous systematic evaluations** throughout the lifecycle of an eHealth intervention. The authors state that RCTs alone fail to take sufficient account of the contextual considerations; these **design methodologies** alone are often less well suited to evaluate the impact of eHealth interventions in a complex environment. According to the authors, design teams need to gain a thorough understanding of the **stakeholders' needs**, concerns, values, and beliefs, and define (as far as possible) what the eventual system will be expected to provide.<br><br><u>Multifaceted, multidisciplined approach</u><br>The authors state that it is important that design teams take a multifaceted and multidisciplined approach to document the complex relationships between the<br>(1) political,<br>(2) social,<br>(3) organizational, and<br>(4) technical worlds.<br>The authors mention the term "key stakeholders", but do not specify who the key stakeholders are.<br><br><u>Continuous systematic evaluations</u><br>Key stages of the eHealth intervention's lifecycle:<br>(1) inception (eg, vision, goals & needs)<br>(2) requirements & analyses<br>(3) design, develop & test<br>(4) implement & deploy<br><br><u>Evaluation methods</u>

		Design methodologies<br>- formative iterative evaluations using simple *prototypes* of the eHealth intervention may be used for requirements elicitation and analyses<br>- once a working model of the system is available, *empirical evaluations* can be completed, which could include the collection of *quantitative and/or qualitative data*, depending on the goals and scope of the study and the stage of development
3	Yusof et al., 2008,<br>An evaluation framework for health information systems: human, organization and technology-fit factors [3]	Structure a debating tool that stakeholders can access in order to know their own health system better. The framework provides **evaluation dimensions** for addressing the fit between human, organization, and technology factors. The **HOT-fit** framework can and should be applied in a flexible way, taking into account different contexts and visions, **stakeholders' point of views**, phases in the system development life cycle, and **evaluation methods**.<br><br><u>Stakeholders participation</u><br>The term "stakeholders" is mentioned, but not specified by the authors.<br>The term "user" refers to:<br>(1) clinicians<br>(2) managers and IT staff<br>(3) system developers<br>(4) hospitals or the entire healthcare sector<br><br><u>Evaluation dimensions (HOT-fit)</u><br>(1) human factors: system use, user satisfaction<br>(2) technology factors: system, information, and service quality<br>(3) organizational factors: structure, environment, communication<br>(4) net benefits: impact on users, performance; efficiency, effectiveness, etc.; organizational impact (eg. costs); clinical impact (quality of life, care, communication/information access).<br><br><u>Evaluation methods</u><br>This framework can be applied using qualitative, quantitative or a combination of both approaches. Methods are presented via a case study:<br>- A formative evaluation was undertaken of the adoption of FIS to identify system problems as they emerged and to improve the system as it was developed.<br>- Qualitative methods were employed to generate a fuller description of the healthcare setting and its cultural issues and to understand why the system functioned well or poorly in a particular setting.<br>- Snowball sampling method was used in order to gain in-depth information from key informants about the development of the FIS.<br>- During observations and face-to-face interviews, individuals including users, clinicians and IT staff that were involved with the system were queried about their system use and patient pathways.
4	Hamid & Sarmad, 2008,<br>Evaluation of e-health services: user's perspective criteria [4]	The framework proposes **user-centered evaluation criteria** for eHealth services. The authors state that the evaluation criteria can serve as part of an eHealth evaluation framework. A **sequential multi-method research approach** is adopted by the authors. The framework only considers one stakeholder or a group of

		stakeholders with a common perspective in an evaluation process; in this study, it is the **user's perspective**.<br><br>User's perspective<br>Users not specified<br><br>User-centered evaluation criteria<br>(1) costs (money and time saving)<br>(2) benefits (effort saving, quality, access)<br>(3) easy to learn/use (to work with a service)<br>(4) accessibility (of content and user interface)<br>(5) compatibility (fit into the healthcare system)<br>(6) functionality (eg, information accuracy, technical functionality)<br>(7) user satisfaction (utility, reliability, efficiency, customization, flexibility)<br><br>Sequential multi-method research approach<br>Methods not mentioned
5	Pagliari, 2007,<br>Design & evaluation in eHealth: challenges and implications for an interdisciplinary field [5]	Framework to facilitate **interdisciplinary collaboration between** software developers and health services researchers. The author discusses the importance of research for ensuring that new eHts are adopted and effective. Evaluation should ideally be approached as a longitudinal process occurring through a series of overlapping and **iterative stages** relevant to the maturity of the technology in its lifecycle, from initial conception to rollout. The framework presents the **evaluation research methods** during the development and implementation process.<br><br>Interdisciplinary collaboration<br>Disciplines:<br>(1) software designers<br>(2) researchers: medical, social, management, legal scientists<br><br>Iterative evaluation stages<br>(1) evaluation of concepts and prototypes (lab): drafting new interventions based on an assessment of stakeholder needs and theory<br>(2) evaluation of impacts: assessing the impact of the innovations on the processes and outcomes of care in selected target settings (experimental studies)<br>(3) pragmatic evaluation: evaluating systems after roll-out (assess impact)<br><br>Evaluation research methods<br>(1) Longitudinal process studies<br>(2) Multiple methods: rigorous qualitative methods (eg, ethnographic studies), and quantitative methods (clinical trials). The author states that controlled trials may be ideal for studying the impact of eHealth systems on measures of clinical outcome or efficiency, but they are poorly suited to exploring social, contextual, or technical barriers to adoption and certainly will have little to offer developers designing a new Web interface. Conversely, think aloud methods may be extremely useful for assessing the usability of a decision-support tool but say very little about its clinical

		validity or effectiveness.
6	Kaufman et al., 2006, Evaluation framework for health information system design, development and implementation [6]	The framework provides a heuristic for matching the stage of system<br>design and the level of evaluation (**continuous evaluation**). A **user-centered approach** to design is presented. The authors state that the incorporation of **sound evaluation methodologies** throughout the stages of system development is necessary to increase the potential of information systems in order to influence healthcare processes and outcomes positively.<br><br><u>User-centered design</u><br>Users: patients and caregivers<br><br><u>Continuous evaluation</u><br>Evaluation activities during stages of system design:<br>(1) specification and needs requirements<br>(2) component development (lab)<br>(3) integration of components in the field<br>(4) integration of system into a clinical setting<br>(5) routine use of a system<br>Design, development and implementation are viewed by the authors as more iterative than sequential activities.<br><br><u>Sound evaluation methodologies</u><br>- Formative methods (eg, needs requirement) are used in the earlier stages<br>- Summative methods to evaluate the validity and efficacy of a system (eg, a controlled clinical trial) are used in the later stages
7	Dansky et al., 2006,<br>A framework for evaluating eHealth research [7]	Holistic framework (template) integration of four **key-dimensions for eHealth evaluation**. The authors state a **multidisciplinary team** is needed and that roles and responsibilities should be identified. The authors suggest combining both quantitative and qualitative **research approaches** to foster a holistic basis for eHealth technologies.<br><br><u>Multidisciplinary development team</u><br>Key stakeholders should participle across the dimensions, communication is the adhesive that holds the framework together (key stakeholders are not specified).<br>Users: an individual, or a community, an organization (not further specified).<br><br><u>Integration of key-dimensions</u><br>(1) research design and methodology (eg, randomization, recruitment strategy)<br>(2) environment (eg, regulations, funding/reimbursement)<br>(3) logistics (eg, roles and responsibilities of a multidisciplinary team, procedures for data collection)<br>(4) technology (eg, technical requirements, infrastructure and resources to support the technology, user issues such as training and satisfaction with the system)<br>The authors suggest that these four dimensions must be integrated to provide a holistic framework for designing and implementing eHealth research projects.

		Quantitative and qualitative research approaches<br>Methods not mentioned; the article does not endorse specific designs, methods, or approaches for conducting eHealth research.
8	Van der Meijden et al., 2003,<br>Determinants of success of inpatient clinical information systems: a literature review (on evaluations of patient care information systems) [8]	The framework proposes **determinants of success** of in-patient clinical information systems. The authors state that the framework is useful in evaluating patient care information systems, with modifications to include contingent factors, such as **user involvement** during system development and implementation and organizational culture. The authors also state that an evaluation should start before the development and should have no fixed end (**continuous formative evaluation**). In evaluations of information systems that employ **multiple methods**, the data from different sources complement each other to provide a more complete picture.<br><br>User involvement<br>Users not specified<br><br>Continuous formative evaluation<br>Start before development, no fixed-end<br><br>Determinants of success<br>(1) system quality attributes (eg, ease of use)<br>(2) information quality attributes (eg, comprehensiveness)<br>(3) individual impact attributes (eg, changed clinical work patterns)<br>(4) usage and user satisfaction attributes (eg, frequency of use, user-friendliness)<br>(5) implementation attributes (eg, communication, training, technical support)<br>(6) organizational impact attributes<br>(7) system development attributes (eg, user involvement)<br>(8) implementation attributes (eg, training)<br>(9) organizational aspects attributes (eg, rewards)<br><br>Multiple methods<br>The integration of qualitative (observations, interviews) and quantitative (questionnaires, work sampling) data collection methods provides an opportunity to improve the quality of the results through triangulation.
9	Shaw, 2002,<br>‘CHEATS’: a generic information communication technology (ICT) evaluation framework [9]	The framework (guideline for gathering information) provides a **comprehensive evaluation strategy** and a **multidisciplinary approach**. The CHEATS framework comprises of six **evaluation aspects** involved in systems design, implementation and use should be taken into account.<br><br>Multidisciplinary development approach<br>Stakeholder groups:<br>(1) caregivers<br>(2) professionals<br>(3) patients<br>(4) client groups (other groups not defined)<br><br>Evaluation aspects<br>(1) clinical (eg, quality of care)

		(2) human & organizational (eg, interface between different healthcare providers)<br>(3) educational (eg, training provision)<br>(4) administrative (eg, cost-effectiveness)<br>(5) technical (eg, ease of use)<br>(6) social (eg, impact on social interaction)<br><br><u>Comprehensive evaluation strategy (methods)</u><br>This involves a continuing process of semi-structured interviews with key participants (**qualitative data**), as well as the collection of **quantitative data**, from questionnaires and existing data, about service use and clinical effectiveness (beyond RCTs, ICT is not a drug and should not be evaluated as such).
10	Kazanjian & Green, 2002, Beyond effectiveness: the evaluation of information systems using a comprehensive health technology assessment framework [10]	The framework provides guidelines for information seeking during development (four **key dimensions**) **for decision-making** about the adoption of health information technologies; identifying stakeholders, needs-assessment (problems, solutions), value specification (beneficiaries, benefits of technology). Identifying relevant interest groups, wider social and political impact of technologies. A **multidisciplinary approach** (inclusion of all stakeholders) is presented.<br><br><u>Multidisciplinary development approach</u><br>Stakeholders:<br>(1) technology producers<br>(2) providers<br>(3) patients and society (primary stakeholders)<br>(4) third-party payers (to know the impact of technology on resource use, implications for accountability)<br><br><u>Key dimensions for decision-making</u><br>(1) population at risk, population impact (disability, quality of life)<br>(2) social context (ethical, legal, political concerns)<br>(3) economic concerns (eg, optimization of total social returns by weighting estimated costs and perceived benefits)<br>(4) technology assessment (eg, increased understanding of conflicting interests)
11	Kushniruk, 2002, Evaluation in the design of health information systems: application of approaches emerging from usability engineering [11]	The framework underlines the importance of evaluation throughout the process of software development (**continual evaluation**). The framework provides **continual evaluation methods** (formative) from project planning to design and implementation.<br><br><u>Continual evaluation</u><br>Evaluation phases:<br>(1) planning (needs analysis; eg, workflow analysis)<br>(2) analysis (requirements, eg, interviews)<br>(3) design (eg, usability testing)<br>(4) implementation (eg, programming, usability testing)<br>(5) support (eg, maintenance; outcome-based evaluations)<br><br><u>Continual evaluation methods</u><br>The framework considers evaluation methods ranging from controlled experimental approaches to naturalistic approaches (ethnographic). Usability testing is presented as a key method for

		conducting evaluations during iterative system development.<br>Integration of data collection from multiple methods (process outcomes + summative outcomes)
12	Hebert, 2001,<br>Telehealth success: evaluation framework development [12]	The framework provides **performance indicators to assess telehealth success**.<br>Similar studies (eg, diabetic homecare) can be examined using the framework to extract commonalities and differences in where telehealth is effective as well as what variables demonstrate “success” (eg, satisfaction).<br><br><u>Performance indicators to demonstrate success</u><br>(1) structure: individual structure patient/provider (eg, access to services; training), organizational structure (eg, cost, culture)<br>(2) process of care: satisfaction, effectiveness, management of the care process<br>(3) individual outcomes (patient/provider, eg, quality of life; number of re-admissions)
13	Eysenbach, 2000,<br>A framework for evaluating eHealth: systematic review of studies assessing the quality of health information and services for patients on the Internet [13]	The framework provides quality indicators for health information and services to patients on the Internet. Quality is classified as **structural quality** (the communication setting, infrastructure, and resources), **process quality** (the communication process itself), and **outcome quality** (the effect of communication).<br><br><u>Quality assessment measures</u><br>(1) Structural quality:<br>- real structure (information providers; criteria; resources, staff, training, internal operating procedures)<br>- virtual structure; Internet venues; system criteria<br>(2) Process quality: communication process (quality of advice and support given; criteria: accuracy, ethical in line with clinical guidelines; privacy, confidentiality, validity of content/tools)<br>(3) Outcome quality: effect of communication (users, patients; eg, quality of life, cost-effectiveness, behavior change)
14	Eng et al., 1999,<br>Evaluation framework for interactive health communication applications [14]	The framework describes criteria for evaluation activities and methods in the eHt development cycle. **Key principles for evaluation** and quality improvement issues for eHts are presented that should be addressed by stakeholders. Four **stakeholder groups must participate** if meaningful evolution and quality improvement of IHC is to occur. The authors state that **evaluation methods** should be woven throughout the conceptualization, design, implementation, and dissemination phases of product development.<br><br><u>Stakeholder participation</u><br>Stakeholder groups that should participate:<br>(1) consumers (patients, families, caregivers)<br>(2) healthcare professionals and purchasers<br>(3) IHC developers<br>(4) policy-makers<br><br><u>Continuous evaluation</u><br>Evaluation phases:<br>(1) conceptualization (f*ormative* evaluation; problems, needs, business plan development, system requirements specification)<br>(2) design (eg, prototyping)

		(3) implementation (*process* evaluation; operational activities, security, reliability, usability, user satisfaction, utilization patterns)<br>(4) assessment & refinement (eg, *outcome* evaluation; revise program, evaluation results)<br><br>Key principles for evaluation<br>(1) evaluation should be practical (methods)<br>(2) evaluation should be pro-active<br>(3) evaluation should have a clear purpose<br>(4) evaluation should be a shared responsibility<br>(5) evaluation should be ubiquitous in product development<br><br>Evaluation methods<br>Active and flexible models of evaluation; the authors mention different methods like focus groups, surveys, interviews, literature review etc.
15	Jai Ganesh, 2004, eHealth - drivers, applications, challenges ahead and strategies: a conceptual framework [15]	The conceptual framework proposes **key-enablers for successful deliverance of e-health services**; the author states that eHealth programs should be based on a sound economic framework and deliver significant value for the investment. **User-centered design** is advantageous to provide services that are valuable to users. **Multidisciplinary collaboration** is necessary to assist in the development of effective and sustainable eHealth programs.<br><br>User-centered design<br>Users are defined as key healthcare players (the health triangle):<br>(1) patients<br>(2) practitioners: any healthcare professional eg, general practitioner or specialist<br>(3) providers: healthcare service providers (eg, hospitals, medical and academic research institutions), diagnostic equipment providers, informatics and computer suppliers, professional associations, health management organizations, insurance companies, the Ministry of Health, pharmaceutical companies<br><br>These key healthcare players should work together to develop, promote and deliver healthcare services. Technology is the linking factor between these key players.<br><br>Multidisciplinary collaboration<br>Disciplines/stakeholder groups:<br>(1) information technology experts<br>(2) health professionals<br>(3) lawyers<br>(3) industry<br>(4) others (not specified)<br><br>Key enablers for successful deliverance of eHealth services<br>(1) defining eHealth needs (needs driven assessment)<br>(2) developing infrastructure requirements<br>(3) mobilizing organizational support<br>(4) planning technically feasible and medically valid applications<br>(5) conducting pilot projects<br>(6) benchmarking successful delivery models

		(7) promoting partnerships
16	Kukafka et al., 2003, Grounding a new information technology implementation framework in behavioral science: a systematic analysis of the literature on IT use [16]	The integrative framework guides IT-implementation plans via a multifactor problem-driven and phased approach. The application of the framework rests on two propositions:<br>(1) IT use is complex, multi-dimensional, and influenced by a variety of factors at individual and organizational levels<br>(2) Success in achieving change is enhanced by the **active participation of members from the target user groups**; to this end the framework promotes participatory design through a linkage system of **critical assessment phases** to ensure that planners have a structure in place to engage end-users effectively from the start.<br><br><u>Active participation of members of the target user group (participatory design)</u><br>The authors state that end-users (not specified) management, and administrators should be engaged as active partners in “diagnosing” the problem. This process enables planners to expand their knowledge of the organization by identifying the values and subjective concerns key stakeholders have with existing systems and procedures. The authors mention the term “key stakeholders”, but do not specify who the key stakeholders are.<br><br><u>Critical assessment phases</u><br>(1) assessment of the organizational needs and goals<br>(2) assessment of organizational needs and goals amenable to IT system solutions<br>(3) identification of behaviors linked with system use<br>(4) assessment of multi-dimensional factors that influence usage behaviors:<br>- predisposing factors (eg, ease of use)<br>- enabling factors (eg, resources, policies)<br>- reinforcing factors (eg, rewards)<br>(5) system use-inducing strategies, focuses on developing and implementing approaches that are proactive and specifically targeted to influencing favorably the predisposing, enabling, and reinforcing factors identified in Phase 4.<br><br><u>Participatory design</u><br>The framework promotes participatory design through a linkage system of critical assessment phases to ensure that the planners have a structure in place to engage system end-users effectively from the start. Methods are not presented.

References

1. Esser P, Goossens R. A framework for the design of user-centred teleconsulting systems. J Telemed Telecare 2009;15(1):32-39. [PMID:19139218]
2. Catwell L, Sheikh A. Evaluating eHealth interventions: the need for continuous systemic evaluation. PLoS Med 2009;6(8):e1000126. [PMID:19688038]
3. Yusof MM, Kuljis J, Papazafeiropoulou A, Stergioulas LK. An evaluation framework for health information systems: human, organization and technology-fit factors (HOT-fit). Int J Med Inform 2008;77(6):386-398. [PMID:17964851]

4. Hamid A, Sarmad A. Evaluation of e-health services: user's perspective criteria. Transforming government: people, process and policy 2008;2(4):243-255. [DOI:10.1108/17506160810917945]
5. Pagliari C. Design and evaluation in eHealth: challenges and implications for an interdisciplinary field. J Med Internet Res 2007;9(2):e15. [PMID:17537718]
6. Kaufman D, Roberts WD, Merrill J, Lai TY, Bakken S. Applying an evaluation framework for health information system design, development, and implementation. Nurs Res 2006;55(2 Suppl):S37-42. [PMID:16601633]
7. Dansky K, Thompson D, Sanner T. A framework for evaluating eHealth research. Eval Program Plann 2006;29(4):397-404. [PMID:17950868]
8. Van der Meijden MJ, Tange HJ, Troost J, Hasman A. Determinants of success of inpatient clinical information systems: a literature review. J Am Med Inform Assoc May-Jun 2003;10(3):235-243. [PMID:12626373]
9. Shaw NT. CHEATS: a generic information communication technology (ICT) evaluation framework. Comput Biol Med 2002;32(3):209-220. [PMID:11922936]
10. Kazanjian A, Green CJ. Beyond effectiveness: the evaluation of information systems using A Comprehensive Health Technology Assessment Framework. Comput Biol Med 2002;32(3):165-177. [PMID:11922933]
11. Kushniruk A. Evaluation in the design of health information systems: application of approaches emerging from usability engineering. Comput Biol Med May 2002;32(3):141-149. [PMID:11922931]
12. Hebert M. Telehealth success: evaluation framework development. Stud Health Technol Inform 2001;84(Pt 2):1145-1149. [PMID:11604908]
13. Eysenbach G. A framework for evaluating e-health: systematic review of studies assessing the quality of health information and services for patients on the Internet. J Med Internet Res 2000;2(suppl2) [DOI:10.2196/jmir.2.suppl2.e13]
14. Eng TR, Gustafson DH, Henderson J, Jimison H, Patrick K. Introduction to evaluation of interactive health communication applications. Science Panel on Interactive Communication and Health. Am J Prev Med Jan 1999;16(1):10-15. [PMID:9894549]
15. Jai Ganesh A. E-health - drivers, applications, challenges ahead and strategies: a conceptual framework. Ind J Med Inform 2004;1:39-47.
16. Kukafka R, Johnson SB, Linfante A, Allegrante JP. Grounding a new information technology implementation framework in behavioral science: a systematic analysis of the literature on IT use. J Biomed Inform 2003;36(3):218-227. [PMID:14615230]

Note. The framework numbers 1-16 correspond as follows to the reference numbers of the manuscript:

Framework	Reference	Corresponding author
fr.1	[104]	Esser & Goossens
fr.2	[23]	Catwell & Sheikh
fr.3	[28]	Yusof et al.
fr.4	[50]	Hamid & Sarmad
fr.5	[48]	Pagliari
fr.6	[29]	Kaufman et al.
fr.7	[6]	Dansky et al.
fr.8	[30]	Van der Meijden et al.
fr.9	[27]	Shaw
fr.10	[49]	Kazanjian & Green
fr.11	[60]	Kushniruk
fr.12	[33]	Hebert
fr.13	[117]	Eysenbach
fr.14	[51]	Eng et al.
fr.15	[52]	Jai Ganesh
fr.16	[26]	Kukafka et al.
