# Supplementary material for: A Holistic Framework to Improve the Uptake and Impact of eHealth Technologies
Source: J Med Internet Res. 2011 Dec 13;13(4):e111. doi: 10.2196/jmir.1672 (PMC3278097; doi:10.2196/jmir.1672)
Supplement: Supplementary file 4 [file jmir_v13i4e111_app4.pdf]

Multimedia Appendix 4. Case studies

The framework is currently being applied and tested in the following research projects of the Center for eHealth Research:

EurSafety Health-net

The European Union wants to optimize the mobility and safety of European patients in the form of adequate cross-border healthcare. However, differences in quality between cross-border healthcare remain problematic. The EurSafety Health-net project has one goal: to improve cross-border cooperation and reduce healthcare-associated infections (HCAIs) with extra attention for multi-resistant pathogens. To achieve this goal, we co-created with all stakeholders a Health-net; a multidisciplinary, Internet-based platform to facilitate this cooperation by allowing professionals to communicate with each other and share infection-related information. The platform will take shape by researching and identifying the necessary evidence-based (expert) content, and also the indispensible, practice-driven structure. This project is using the CeHRes Roadmap to guide the development of the Internet-based platform.

DiaDerma

DiaDerma is a project to develop a tele-diagnostic device for the effective screening and monitoring of chronic wounds on the skin. Most chronic wound diagnoses are made in the home and often these diagnoses are not optimal. The device shall help home-based caregivers to make a snapshot of the wound for an eventual automated diagnosis with a chronological collection of snapshots to see how the wound evolves. We used our framework to identify the problems and specify what the technology wants to achieve (problem statement); to perform a stakeholder analysis and needs assessment, to translate the critical values into functionalities of technology (program of requirements), to match user requirements with technical requirements, to set criteria for resources and competences, to test the scenarios for using the technology in different care settings and to make a business model.

Diabetes Interactive Education Program

The Diabetes Interactive Education Program (DIEP) is an online application for people with type 2 Diabetes Mellitus. It has been developed to support patient education and stimulate self-management. Though rated by experts and patients as one of the best interventions available in the Netherlands today, some deficiencies impede its nationwide implementation among a wide range of people. To remove these deficiencies we re-designed DIEP using a collaborative approach. Using concepts and techniques from participatory (re)design and business modelling we determined the user needs (patients, health care providers, and other stakeholders) and define what should be done to improve usability, adherence, and acceptance. The outcome is an optimized DIEP; a persuasive tool that people use and keep using since is serves the goals they have set and helps them to cope with diabetes.
